# Tylectomy Safety in Salvage of Eyes with Retinoblastoma

**DOI:** 10.3390/cancers13225862

**Published:** 2021-11-22

**Authors:** Junyang Zhao, Qiyan Li, Zhao Xun Feng, Jianping Zhang, Songyi Wu, Liwen Jin, Brenda L. Gallie

**Affiliations:** 1 Department of Ophthalmology, Liuzhou Maternity and Child Healthcare Hospital, Liuzhou 545001, China; zhaojunyang@163.com (J.Z.); 13768340179@163.com (J.Z.); 2 Pediatric Oncology Center, Beijing Children’s Hospital, Beijing 100045, China; 3 Department of Ophthalmology, Beijing Tongren Hospital, Beijing 100730, China; li_qiyan@sina.com.cn; 4 Department of Ophthalmology, University of Ottawa, Ottawa, ON K1L 8L6, Canada; zfeng@toh.ca; 5 Quanzhou Aier Eye Hospital, Quanzhou 362017, China; 13808520745@139.com (S.W.); fxjlw@163.com (L.J.); 6 Department of Ophthalmology and Vision Science, Hospital for Sick Children, Toronto, ON M5G 1X8, Canada; 7 Krembil Research Institute and Techna Institutes, University Health Network, Toronto, ON M5T 2S8, Canada; 8 Departments of Ophthalmology and Vision Science, Molecular Genetics, and Medical Biophysics, University of Toronto, Toronto, ON M5T 3A9, Canada

**Keywords:** retinoblastoma, tylectomy, pars plana vitrectomy, surgery, resection, endoresection, survival, safety, enucleation

## Abstract

**Simple Summary:**

The role of organ-conserving surgery has not been explored in retinoblastoma as it has been in other cancers, such as breast cancer lumpectomy, partial nephrectomy for kidney cancer, and partial orchiectomy for testis cancer. This is largely accounted for by the high mortality of extraocular retinoblastoma compared to intraocular retinoblastoma, and fear of iatrogenic tumor spread with intraocular surgery. We propose the little-known word “tylectomy” (“tulos”, Greek for “lump”) to describe the surgical resection of retinoblastoma. Through review of consecutive patients treated by our team between 2012–2014, we compared survival of patients with eye salvage, including tylectomy, to those who had eye salvage without tylectomy or primary enucleation. We found that patients who had tylectomy had superior survival compared to those who had eye salvage without tylectomy (96% vs. 90%), and comparable survival to those with primary enucleation (96% vs. 95%). Our study supports tylectomy as a safe contribution to retinoblastoma management.

**Abstract:**

Intraocular surgery is tabooed in retinoblastoma management, due to the concern of lethal extraocular spread. We reviewed the outcomes of consecutive children with intraocular retinoblastoma diagnosed at 29 Chinese centers between 2012–2014. We compared the outcomes of three categories of treatment: eye salvage including tylectomy (Group I), eye salvage without tylectomy (Group II), and primary enucleation (Group III). A total of 960 patients (1243 eyes) were diagnosed: 256 in Group I, 370 in Group II, and 293 in Group III; 41 patients abandoned treatment upfront. The estimated 5-year overall survivals (OS) were, for Group I, 94%, for Group II 89%, and for Group III 95%. The estimated 5-year disease-specific survivals (DSS) were, for Group I, 96%, for Group II 90%, and for Group III 95%. Patients in Group I had a significantly higher 5-year DSS than patients in Group II (*p* = 0.003) and not significantly different than patients in Group III (*p* = 0.367). Overall survival was not compromised by the inclusion of tylectomy in eye salvage therapy compared to eye salvage without tylectomy or primary enucleation. Disease-specific survival was better when tylectomy was included in eye salvage treatments. Tylectomy as part of multimodal treatment may contribute to the care of retinoblastoma patients with chemotherapy-resistant tumor, eyes with concomitant ocular complications, or at the risk of treatment abandonment.

## 1. Introduction

The high mortality of extraocular retinoblastoma compared to primary enucleation led to the dogma that intraocular procedures have no role in eyes with retinoblastoma [1]. This was successfully challenged by planned intravitreal pars plana injections demonstrating success in treating vitreous seeds with low risk (<1%) of extraocular tumor spread [2]. Considering the efficacy of intravitreal melphalan [3,4] and technical advances in small gauge vitrectomy [5], we incorporated retinoblastoma resection in multimodal retinoblastoma management [6].

Modern cancer practice has embraced organ conserving surgery, such as breast cancer lumpectomy [7], partial nephrectomy for kidney cancer [8,9], and partial orchiectomy for testis cancer [10,11]. While lumpectomy officially refers only to breast cancer, the little-known word “tylectomy” refers to surgical removal of a tumor (“tulos”, Greek for “lump”). We propose the word tylectomy to describe organ-conserving surgical resection of retinoblastoma.

Despite attempted eye salvage with combinations of chemotherapy (systemic, intra-arterial, and intravitreal), refractory disease and tumor recurrence remain common. These eyes often necessitate enucleation to save the child’s life. In contrast to traditional eye salvage modalities, tylectomy surgically removes the tumor irrespective of chemotherapy-resistance. Tylectomy therefore presents an additional opportunity for salvage of eyes that failed chemotherapy. 

Moreover, treatment abandonment is a leading cause of retinoblastoma mortality in low- and middle-income countries [12,13]. The combinations of social, cultural, and financial factors may lead families in these settings to refuse enucleation despite the mortality risk of active retinoblastoma [14]. In these difficult situations, tylectomy presents an additional eye salvage opportunity for patients who failed conventional therapies. Families who cannot afford successive sessions of chemotherapy or extended follow-up may also elect for tylectomy. Although loss to follow-up post-surgery is suboptimal, it may achieve a better outcome than upfront treatment abandonment with 100% mortality [15]. 

The main concern with intraocular surgery in eyes with retinoblastoma is the risk of iatrogenic extraocular spread. Our prior case series suggested a role for resection of refractory retinoblastoma [6], but safety concerns remained given the small sample size. Intraocular surgery in eyes with unsuspected retinoblastoma has been associated with metastatic disease [16,17], but metastasis was not identified in case reports of planned tumor excision [6,18,19,20,21,22]. The present study offers a macroscopic overview of the safety of tylectomy relative to conventional treatments. The primary objective is to determine the overall survival (OS) and disease-specific survival (DSS) of three groups of patients who received eye salvage therapies, including tylectomy, eye salvage therapies without tylectomy, or primary enucleation.

## 2. Materials and Methods

### 2.1. Data Collection and Ethics

Retrospective data of consecutive patients with intraocular retinoblastoma diagnosed at 29 Chinese hospitals between 2012 to 2014 were reviewed. The date of the last follow-up was 1 February 2021. A retrospective review of medical records without research consent was approved by the Ethics Boards of Liuzhou Maternity and Child Healthcare Hospital in accordance with the Declaration of Helsinki. 

The collected clinical information included age at diagnosis, sex, disease laterality, International Intraocular Retinoblastoma Classification (IIRC, Table S1) [23] clinical eye stage, pTNM 8th edition histopathologic staging [24], type and regimen of chemotherapy, cause of death, and dates of diagnosis, chemotherapy, tylectomy, recurrence, enucleation, last follow-up, and death.

### 2.2. Treatment

Group I patients received tylectomy (in addition to other salvage treatments). Group II patients received salvage treatments, but not tylectomy. Group III patients received primary enucleation without attempted salvage treatment. Patients who had metastatic death are grouped based on treatments of the eye that likely caused mortality. When the eye responsible for metastatic death is unknown, death is attributed to the tylectomy eye. For bilateral patients with both eyes in the same treatment group, clinical information of the right eye is used for analysis. 

The standard-of-care follow-up interval was 1–2 months during active treatment. Children with no active tumor after treatment were followed every month for the first 4 months, every 2 months for the next 8 months and then longer intervals. Follow-up involved fundoscopic examination, evaluation by medical oncology, and imaging if extraocular extension was suspected. Lost to follow-up is defined as absence at the latest scheduled appointment and inability to contact the parents.

All retinoblastoma children in this study were managed by one retinoblastoma team led by J.Z. (retinoblastoma specialist) under uniform protocol. J.Z. routinely traveled to the 29 hospitals and was the most responsible physician for retinoblastoma children managed at these centers. Children with indications for tylectomy were referred, with consent, to three centers that have the authorization for tylectomy: Liuzhou Maternity and Healthcare Hospital, Quanzhou Aier Eye Hospital, and Beijing Tongren Hospital. All tylectomies were performed by J.Z. (retinoblastoma specialist) and Q.L. (vitreoretinal surgeon).

**Figure 1:**
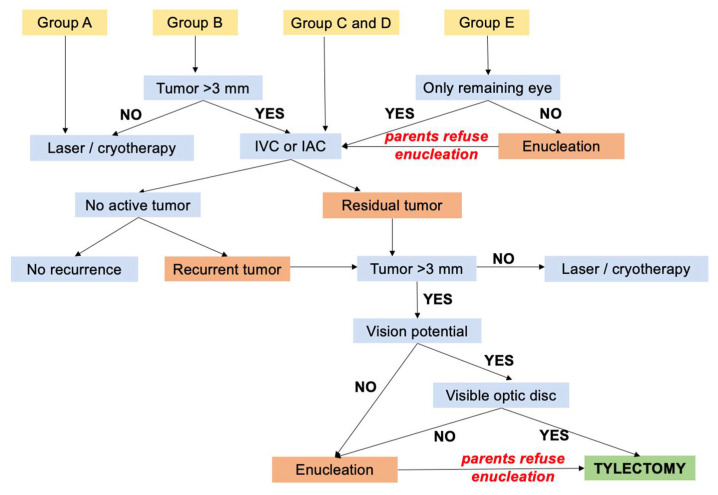
Standard care for retinoblastoma by IIRC Group leading to eligibility for tylectomy. IVC, intravenous chemotherapy; IAC, intra-arterial chemotherapy.

The standard management leading to tylectomy is summarized in Figure 1. Tylectomy was primarily used as a secondary treatment for residual tumor or recurrence following systemic or intra-arterial chemotherapy. Tylectomy was considered for these circumstances: following chemotherapy, lack of significant regression, progression, new tumor activity, new subretinal, epiretinal or vitreous seeds, opacities obscuring tumor visualization; parent preference; or refusal of enucleation. All options including enucleation were presented to the family and informed consent was obtained prior to the tylectomy. Following tylectomy, adjuvant intravitreal chemotherapy was offered to patients with diffuse intravitreal seeds and adjuvant systemic chemotherapy was offered to patients with extensive retinal invasion or suspected choroidal invasion, discovered intraoperatively. 

### 2.3. Indications and Contraindications

All patients were screened for extraocular disease at diagnosis and/or prior to tylectomy by magnetic resonance imaging (MRI). Absolute contraindications to tylectomy were evidence of optic nerve or extrascleral invasion. Relative contraindications were obscured optic disc, foveal invasion, or extensive retinal invasion requiring large retinal resection. The decision to proceed with tylectomy for each patient was based on the clinical features and parents’ choice. For Group E, eyes (advanced intraocular retinoblastoma) enucleation was presented to parents as the safest treatment option. 

### 2.4. Procedure

Under general anesthesia, a dilated fundus exam was performed with scleral depression. Tumor-free sclerotomy sites were identified. In eyes with poor fundus visualization due to vitreous opacities, ultrasound biomicroscopy or B-scan ultrasonography were used to survey sclerotomy sites. To reduce the risk of retinal detachment, laser barrier scars were placed around tumors prior to and during surgery.

Standard three-port 23- or 25-gauge non-valved trocar/cannulas were inserted transconjunctivally at pre-selected sites. Melphalan (5 μg/mL) in a balanced salt solution (dose nontoxic to retina in animal studies [25]) was infused continuously throughout the surgery and irrigated onto the ocular surface every 3–5 min. Using a vitrector, the vitreous seeds were aspirated, and soft tumors were endoresected and aspirated. Any visible tumor that extended into the choroid was resected down to the bare sclera via vitrector, after endodiathermy to occlude choroidal vasculature surrounding the tumor. Lensectomy was performed if cataract precluded visualization, or anterior segment tumor seeds were aspirated, or invaded ciliary body was resected. Residual calcified tumors were disrupted by a fragmatome through 20-gauge sclerotomies, and either aspirated or removed from sclerotomy sites using forceps. 

Silicone oil was placed after the tylectomy to stabilize residual retina whenever retinal detachment was threatened, as in partial retinectomy > 3 mm or anterior resection. After the tylectomy, the scleral surface was exposed with peritomy and washed with a 5 μg/mL melphalan irrigation fluid. Vitrectomy port sites, tenon’s capsule, and conjunctiva were sutured. At end of the surgery, 0.2 mL melphalan (5 μg, 25 μg/mL) was injected subconjunctivally at the port sites. 

### 2.5. Statistical Analysis

Sex, age at diagnosis, laterality, follow-up, IIRC Group, time from diagnosis to tylectomy/enucleation, chemotherapy cycles, and AJCC histopathology were summarized using frequency/percentage for categorical variables and median/range for continuous variables. Continuous variables were compared between study groups via the Mann–Whitney U test. The Kaplan–Meier method was used to estimate the OS and DSS; a pairwise comparison was performed between the study groups. The OS accounts for mortality due to any cause, while the DSS accounts only for mortality due to retinoblastoma metastasis. Patients were censored at the last follow-up. All *p*-values reported are two sided; *p* < 0.05 indicated significance. All analyses were performed using SPSS Version 25 (IBM Corp, New York, NY, USA).

## 3. Results

### 3.1. Baseline Data

A total of 960 patients (1243 eyes) were diagnosed with intraocular retinoblastoma in the studied period (Table S2; raw data). Patients in this study included 256 (26.7%) in Group I (tylectomy group), 370 (38.5%) in Group II, 293 (30.5%) in Group III; 41 (4.3%) patients did not receive any treatment due to upfront treatment abandonment. The baseline characteristics of the study patients are presented in Table 1. 

### 3.2. Group I Treatment and Ocular Outcomes

Of the 256 patients in Group I, 11 (4%) received primary tylectomy and 245 (96%) received secondary tylectomy; 31 patients had bilateral tylectomy. The indications for those who received primary tylectomy were vitreous opacity concomitant with active retinoblastoma (2; patients #6 and #7), diagnostic clarification for eyes with media opacity from corneal opacity, dense cataract, hyphema, hypopyon or vitreous hemorrhage (4; patients #180, #197, #251, and #274), and parental refusal of chemotherapy and enucleation (5; patients #257, #265, #634, #670, and #707).

For those who received secondary tylectomy, the main pre-tylectomy treatments were laser photocoagulation (2), IVC (196), IAC (15), and a combination of IVC and IAC (32). Pre-tylectomy, chemotherapy patients received IVC median 3 cycles (range, 1–12 cycles) and/or IAC 2 cycles (range, 1–9 cycles). Other ancillary pre-tylectomy treatments included intravitreal chemotherapy (14), external beam radiotherapy (1), and I^125^ plaque radiotherapy (1). The time from diagnosis to tylectomy was median 4 months (range, 0–50 months). 

Following tylectomy, 127 (50%) patients received adjuvant treatment. Post-tylectomy adjuvant treatments included IVC alone (40), intravitreal chemotherapy alone (52), or a combination of IVC and intravitreal chemotherapy (35). Adjuvant therapies were IVC median 1 cycle (range 1–7 cycles) and intravitreal chemotherapy median 1 cycle (range 1–6 cycles). 

**Figure 2:**
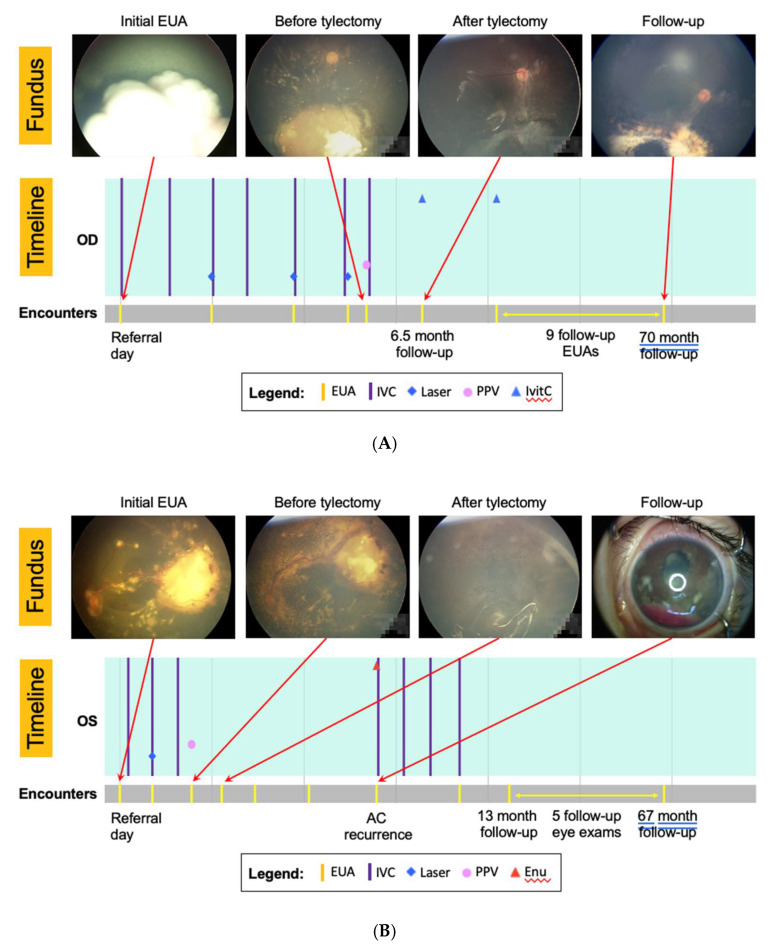
(**A**) Images and treatment timeline of patient #505 treated with tylectomy for refractory tumor who achieved good visual outcome. The patient was diagnosed with unilateral Group D retinoblastoma in the right eye at 2 years of age. Following 6 cycles of systemic chemotherapy, there was still a significant active tumor inferior to the optic disc and vitreous seedings. A tylectomy was performed to remove all active tumors inside the eye. One cycle of adjuvant systemic chemotherapy was given 2 days following tylectomy. There was no tumor recurrence at 70-month follow-up with visual acuity tested 20/100. IVC, intravenous chemotherapy; Ivitc, intravitreal chemotherapy. (**B**) Images and treatment timeline of patient #607 treated with tylectomy for refractory tumor who developed anterior chamber tumor recurrence. The patient was diagnosed with unilateral Group D retinoblastoma in the left eye at 2 years of age. Following 3 cycles of systemic chemotherapy, there was a persistent large active tumor in the temporal retina, diffuse subretinal seedings, and serous retinal detachment. The parents declined enucleation. A tylectomy was performed to remove all active tumors inside the eye. Five months following tylectomy, the eye developed anterior chamber tumor recurrence. The eye was enucleated and had pT3a histopathology. The child received 4 cycles of systemic chemotherapy. There was no evidence of orbital recurrence at 67 months follow-up. IVC, intravenous chemotherapy; Enu, enucleation; AC, anterior chamber.

Tumor recurrence developed in 75 (29%) patients post-tylectomy (Table S3). Locations of recurrence were peripheral retina (40), posterior pole (13), optic nerve head (3), posterior chamber (4), anterior chamber (13), and concomitant anterior chamber and posterior pole (2). Following recurrence, 52 eyes received additional eye salvage treatments. Details of recurrence treatment after tylectomy are presented in Table S3. Overall, 52 (20%) Group I eyes were enucleated, and the estimated 5-year eye salvage rate was 78.9% (95% CI: 73.7%–84.1%). Clinical images of an eye with refractory tumor successfully salvaged following tylectomy (patient #505; Figure 2A), and an eye enucleated 5 months after tylectomy (pT3a), for anterior chamber recurrence (patient #607; Figure 2B) are shown.

### 3.3. Group II Treatment and Ocular Outcomes

Of the 370 patients in Group II, the main eye salvage therapies were laser photocoagulation or cryotherapy (25), IVC (255), IAC (30), and a combination of IVC and IAC (60). The patients received median IVC 3 cycles (range, 1–12 cycles) and/or IAC 2 cycles (range, 1–7 cycles). Other ancillary eye salvage therapies included intravitreal chemotherapy (14), external beam radiotherapy (2), and I^125^ plaque radiotherapy (1). Overall, 175 (47%) Group II eyes were enucleated, and the estimated 5-year eye salvage rate was 42.1% (95% CI: 36.4%–47.8%). 

### 3.4. Group III Treatment and Ocular Outcomes

Of the 293 patients in Group III, 100% of eyes were primarily enucleated without other treatment; 1 patient had bilateral primary enucleation. Median time from diagnosis to enucleation was 0 months or same day enucleation (range, 0–21 months). 

### 3.5. Survival Analysis

**Figure 3:**
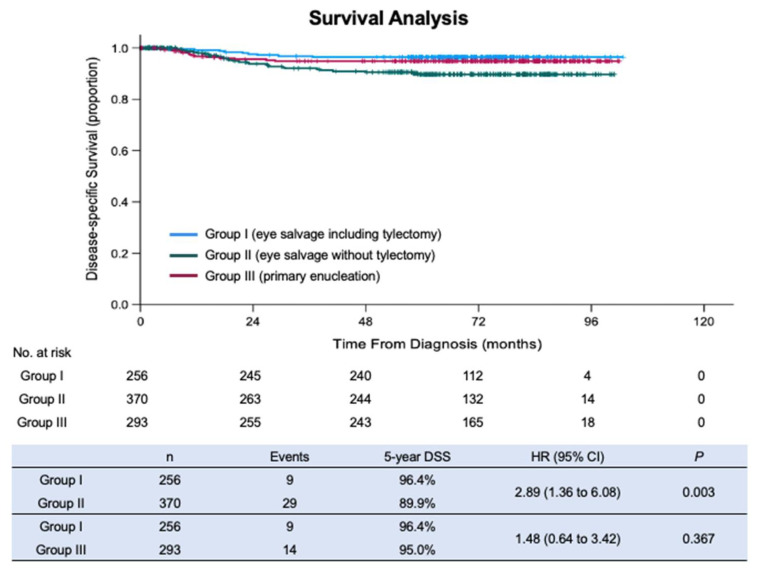
Kaplan–Meier curves of disease-specific survival (DSS) of patients from Group I, II, and III.

For the 59 patients who died, cause of death was retinoblastoma metastasis (52), pinealoblastoma (4), intracranial hemorrhage (1), brain stem glioma (1), and acute lymphocytic leukemia (1). The estimated 5-year OS was, for Group I, 93.9% (95% CI: 90.3%–97.4%), for Group II 88.9% (95% CI: 86.4%–92.6%), and for Group III 95.0% (95% CI: 92.3%–97.6%). The estimated 5-year DSS was, for Group I, 96.4% (95% CI: 94.1%–98.8%), for Group II 89.9% (95% CI: 85.9%–93.5%), and for Group III 95.0% (95% CI: 92.3%–97.6%) (Figure 3). The patients in Group I had a significantly higher DSS than the patients in Group II (*p* = 0.003) and not significantly different from the patients in Group III (*p* = 0.367). The patients in Group I were 2.9 times more likely to survive than the patients in Group II (HR 2.89; 95% CI: 1.35–6.08). The patients in Group III also had a significantly higher DSS than the patients in Group II (*p* = 0.038).

### 3.6. Analysis of Patients Who Died

Nine patients from Group I died of retinoblastoma metastasis, one after primary tylectomy, and eight after secondary tylectomy following IVC or IAC. The locations of intraocular tumor recurrence following tylectomy were posterior pole (2), peripheral retina (7), and none had clinically evidence recurrence at the sclerotomy sites. The locations of the initial metastasis were orbit (6), brain (2), and lung and bone (1). The median times from diagnosis to metastasis and death were 17.8 months (range, 3.7–26.1 months) and 21.8 months (range, 4.7–36.7 months), respectively. None of these nine patients received post-tylectomy adjuvant treatment and five out of nine patients refused enucleation despite uncontrolled tumor recurrence.

Twenty-nine patients from Group II died of retinoblastoma metastasis. The locations of the initial metastasis were orbit (8), brain (7), cerebrospinal fluid (1), skull (1), abdomen (1), neck (1), brain and orbit (1), brain and skull (1), skull, mouth, and abdomen (1), and unknown (7). The median times from diagnosis to metastasis and death were 17.6 months (range, 3.8–41.5 months) and 19.8 months (range, 7.6–59.1 months), respectively. Of the 29 patients, 16 refused enucleation despite uncontrolled tumor recurrence. 

Fourteen patients from Group III died of retinoblastoma metastasis. The locations of the initial metastasis were orbit (4), brain (4), skull (1), lumbar spine (1), brain and cerebrospinal fluid (1), and unknown (3). The median times from diagnosis to metastasis and death were 7.5 months (2.8–24.3 months) and 10.7 months (4.3–28.7 months), respectively.

## 4. Discussion

Our study evaluated the survival of 960 consecutive retinoblastoma patients managed by our retinoblastoma team with and without tylectomy. We report that the inclusion of tylectomy as a component of eye salvage therapy to maximize the functional and cosmetic outcomes of eyes affected by retinoblastoma does not compromise survival. The patients who had tylectomy (Group I) had superior survival compared to those who had eye salvage without tylectomy (Group II; 5-year DSS 96.4% v 89.9%; *p* = 0.003) and the survival was not inferior to those with primary enucleation (Group III; 5-year DSS 96.4% v 95.0%; *p* = 0.367). Currently, three Chinese centers routinely perform tylectomy as part of the multimodal treatment of retinoblastoma. The concern that tylectomy could increase mortality relative to other treatments was not validated.

To the best of our knowledge, this is the first study to evaluate survival outcome of patients with retinoblastoma following planned intraocular surgery. In a case series of 14 patients, Kaliki et al. showed that 8/14 (57%) patients died following intraocular surgery in children with unsuspected retinoblastoma [17]. Unlike our cohort, diagnosis of retinoblastoma was incidental in these 14 children and retinoblastoma-specific safety precautions (e.g., chemotherapy infusion) were not taken during surgery. Furthermore, 11/14 (79%) patients had extraocular tumor extension at initial presentation to ocular oncology clinic. In contrast, the tylectomies in our cohort were planned with the intension of tumor resection and all patients had intraocular disease at diagnosis.

Survival in children with retinoblastoma is influenced by clinical, social, and cultural factors. In a multicenter collaborative study, Tomar et al. showed that 5-year estimated overall survivals were 99% in high-income countries, 89% in upper middle-income countries, and 90% in lower middle-income countries [26]. Given that national income level, a surrogate for healthcare resources, is recognized to have a profound effect on patient survival, it is important that our study compared the survival of patients managed by one retinoblastoma team under a uniform protocol. In comparison, in the AHOPCA II multicenter study of Central American patients with unilateral retinoblastoma (International Retinoblastoma Staging System Stage I; eye enucleated, completely resected histologically), the 102 patients who received primary enucleation had a 5-year overall survival rate of 94% [27]. Furthermore, a Chinese multicenter study reported a 5-year overall survival rate for unilateral and bilateral children treated with primary enucleation of 90% and 87%, respectively [15]. Primary enucleation is generally considered the safest treatment modality for retinoblastoma. In our cohort, patients with tylectomy as part of multimodal treatment demonstrated non-inferior survival compared to those treated with primary enucleation, supporting tylectomy as a safe inclusion in retinoblastoma care. We considered that tylectomy for retinoblastoma could be a viable eye salvage option because of two major advances in retinoblastoma care. First, since injection of melphalan into the vitreous is now a standard in the treatment of retinoblastoma [3,4], during tylectomy a non-toxic concentration of melphalan was continuously infused into the eye and periodically irrigated onto the ocular surface. Second, the small gauge non-valved cannula avoids intraocular pressure increase and directs irrigation fluid to the outer conjunctival surface where melphalan irrigation is applied to reduce the risk of subconjunctival and scleral wall seeding. 

Chemotherapy resistance is a common cause for eye salvage to fail. In a meta-analysis of 1483 eyes that received systemic chemotherapy (IVC), the eye salvage rates were 40% for Group D eyes and 19% for Group E eyes [28]. In a systemic review of 757 eyes, intra-arterial chemotherapy (IAC) achieved an eye salvage rate of 66% [29]. The failure of attempted eye salvage arises from recurrence of chemotherapy-resistant tumor, or intraocular complications, such as cataract, retinal detachment, vitreous hemorrhage, or vitreous seeds that preclude safe monitoring for tumor progression and focal therapy (laser, cryotherapy, and brachytherapy) [30,31]. 

Additional cycles of chemotherapy following initial failure may have decreasing likelihood of success [32,33,34]. In contrast, tylectomy eliminates tumor irrespective of tumor biology. The chemotherapy-induced neutropenia and vitreous opacity (e.g., vitreous hemorrhage) that may preclude eye salvage may be circumvented by tylectomy. Tylectomy was predominantly used as a secondary therapy to chemotherapy. While the focus of this paper is on survival, we noted that the 5-year eye salvage rate of Group I is significantly higher than Group II (78.9% v 42.1%; *p <* 0.001).

There was a considerable variation to the number of cycles of chemotherapy before and after tylectomy because of treatments for the contralateral eye, responses to therapy, and discretion of medical oncology teams at different institutions. Tylectomy was not recommended as a primary therapy, but was performed in selected cases in which an active tumor was present with significant concomitant vitreous opacities and diagnosis of retinoblastoma was uncertain due to media opacity or parental refusal of both chemotherapy and enucleation. 

The anterior segment was a common area for post-tylectomy tumor recurrence, possibly related to limited view and access during vitrectomy to tumor seeds on iris, ciliary body, and zonules. The anterior chamber tumor was excised whenever noted, but residual tumor may have led to some recurrences, as shown in Figure 2B. Furthermore, tylectomy only treats the intraocular space without any extraocular protection. We note that all Group I patients who died of metastases did not receive adjuvant chemotherapy. We hypothesize that subclinical blood metastasis or seeding of the optic nerve, neither which can be managed by tylectomy, may have contributed to the poor outcomes following tylectomy. For these reasons, since 2017, we routinely recommend 2twocycles of adjuvant systemic chemotherapy to all patients following tylectomy.

In low- and middle-income settings, such as China, it is not uncommon for children to abandon treatment for a variety of reasons, including unwillingness to enucleate, financial constraints, long distance from treatment center, and perception that the disease was cured [12,35]. A high proportion parents who refuse enucleation following failed eye salvage with chemotherapy, accept tylectomy as a last resort to save the child’s life and eye. To avoid treatment abandonment, we treated some patients with tylectomy at the parents’ request, despite no possibility of useful vision from the eye. 

The strengths of this study are its large sample size, extended follow-up, and uniform treatment performed by a single highly specialized multidisciplinary team, led by the vitreoretinal surgeon (QL) and retinoblastoma specialist (JZ). This study is limited by the lack of histopathology on material aspirated during tylectomy and the lack of histopathologic evaluation of sclerotomy sites since the standard histopathology sections on enucleated eyes did not contain the sclerotomy sites. This study is also limited by being a retrospective study rather than a randomized controlled trial; treatment selection bias and confounding factors may exist between groups.

For all children with retinoblastoma, the safest approach is primary enucleation. Furthermore, excellent implant motility can be achieved and cosmesis is particularly good in eyes with small palpebral fissure and dark iris. However, when parents choose eye and/or vision salvage over enucleation, tylectomy may be considered as part of multimodal treatment. Absolute contraindications remain MRI evidence of optic nerve invasion or extrascleral extension. Tylectomy of retinoblastoma without careful selection of tumor-free sclerotomy sites, continual melphalan infusion, dedicated retinoblastoma expertise, and fully informed family consent may be dangerous.

In 1989, Fisher et al. showed that lumpectomy with adjuvant irradiation for Stage I and II breast cancer yields non-inferior survival rates compared to mastectomy [7]. Similar to breast cancer lumpectomy, we now show that tylectomy in well-selected patients (radiography excluding extraocular tumor) yielded non-inferior survival rates compared to conventional treatment that includes primary enucleation.

## 5. Conclusions

The 5-year DSS for 256 consecutive patients treated with eye salvage therapies, including tylectomy, was 96.4%, comparable to 95.0% for primary enucleation (*p* = 0.367) and superior to eye salvage without tylectomy (89.9%) (*p* = 0.003). Primary enucleation is commonly regarded as the safest treatment for intraocular retinoblastoma, offered to patients with an eye with advanced retinoblastoma and poor prognosis for useful vision who have a functional contralateral eye. The similar DSS of patients treated with primary enucleation or tylectomy as part of a multimodal treatment indicates that tylectomy is a safe contribution to retinoblastoma eye salvage.

## Figures and Tables

**Table 1:** Baseline clinical characteristics.

Characteristic		Group I (*n* = 256)	Group II (*n* = 370)	Group III (*n* = 293)
Sex				
Male		149 (58%)	198 (54%)	171 (58%)
Female		107 (42%)	172 (46%)	122 (42%)
Age of diagnosis (months)				
Median		20	15	23
Range		1–117	0–119	2–60
	Laterality			
	Unilateral	161 (63%)	211 (57%)	291 (99%)
	Bilateral	95 (37%)	159 (43%)	2 (1%)
Follow-up Time (months)				
	Median	70	62	77
	Range	2–103	0–101	0–102
	Lost to follow-up	5 (2%)	89 (24%)	28 (10%)
IIRC of Studied Eye				
	A	1 (0.4%)	17 (5%)	0 (0%)
	B	8 (3%)	47 (13%)	0 (0%)
	C	15 (6%)	15 (4%)	0 (0%)
	D	180 (70%)	198 (53%)	114 (39%)
	E	52 (20%)	89 (24%)	151 (51%)
	Unknown	0 (0%)	4 (1%)	28 (10%)
Enucleation				
	No	204 (80%)	195 (53%)	0 (0%)
	Yes	52 (20%)	175 (47%)	293 (100%)
AJCC Histopathology				
	pT1	20 (38%)	117 (67%)	146 (50%)
	pT2	10 (19%)	7 (4%)	23 (8%)
	pT3	14 (27%)	18 (10%)	68 (23%)
	pT4	6 (12%)	10 (6%)	10 (3%)
	Unknown	2 (4%)	23 (13%)	46 (16%)

## Data Availability

Raw data is available as Supplementary File.

## Supplementary Materials

The following are available online at https://www.mdpi.com/article/10.3390/cancers13225862/s1: Table S1; Table S2: Raw data, Table S3: Treatments and outcomes of tumor recurrence in Group I.
