# Supplementary material for: Tylectomy Safety in Salvage of Eyes with Retinoblastoma
Source: Cancers (Basel). 2021 Nov 22;13(22):5862. doi: 10.3390/cancers13225862 (PMC8616183; doi:10.3390/cancers13225862)
Supplement: Supplementary file 1 [file cancers-13-05862-s001.zip › Table S1.pdf]

Table S1. International Intraocular Retinoblastoma Classification

Group A (very low risk)	All tumours are 3 mm or smaller, confined to the retina and at least 3 mm from the foveola and 1.5 mm from the optic nerve. No vitreous or subretinal seeding is allowed
Group B (low risk)	Eyes with no vitreous or subretinal seeding and discrete retinal tumour of any size or location. Retinal tumours may be of any size or location not in group A. Small cuff of subretinal fluid extending ≤5 mm from the base of the tumour is allowed
Group C (moderate risk)	Eyes with focal vitreous or subretinal seeding and discrete retinal tumours of any size and location. Any seeding must be local, fine, and limited so as to be theoretically treatable with a radioactive plaque. Up to one quadrant of subretinal fluid may be present
Group D (high risk)	Eyes with diffuse vitreous or subretinal seeding and/or massive, non-discrete endophytic or exophytic disease. Eyes with more extensive seeding than Group C. Massive and/or diffuse intraocular disseminated disease including exophytic disease and >1 quadrant of retinal detachment. May consist of 'greasy' vitreous seeding or avascular masses. Subretinal seeding may be plaque-like
Group E (very high risk)	Eyes that have been destroyed anatomically or functionally with one or more of the following: Irreversible neovascular glaucoma, massive intraocular haemorrhage, aseptic orbital cellulitis, tumour anterior to anterior vitreous face, tumour touching the lens, diffuse infiltrating retinoblastoma and phthisis or pre-phthisis
