# Supplementary material for: Tylectomy Safety in Salvage of Eyes with Retinoblastoma
Source: Cancers (Basel). 2021 Nov 22;13(22):5862. doi: 10.3390/cancers13225862 (PMC8616183; doi:10.3390/cancers13225862)
Supplement: Supplementary file 1 [file cancers-13-05862-s001.zip › Table S2.pdf]

Safety of Tylectomy in Salvage of Eyes Affected by Retinoblastoma
Raw data, Table S2

Patient ID	Group	Sex	Age at Dx months	Studied Eye	IIRC	Follow-up Months	Pre-Tyl IVC #	Pre-Tyl IAC #	Dx to Tyl months	Post-Tyl IVC #	Post-Tyl Ivitc #	Post-Tyl Recur-rence	Enu	Dx to Enu	pTNM	OS	DDS	Dx to Death months
1	2	M	15	OS	E	17	1	0					No			Neg	Neg	17
2	2	M	12	OS	E	59	4	0					Yes	25	pT4	Neg	Neg	59
3	3	F	3	OD	E	81	0	0					Yes	21	pT1	Pos	Pos	
4	2	M	5	OD	E	31	4	0					No			Neg	Neg	31
5	2	M	16	OS	E	58	4	2					Yes	40	pT4	Neg	Neg	58
6	1	F	69	OD	E	66	0	0	0	3	0	Yes	Yes	61	pT4	Pos	Pos	
7	1	M	34	OS	E	5	0	0	0	0	0	Yes	No			Neg	Neg	5
8	1	M	8	OD	E	64	6	0	8	0	0	No	No			Pos	Pos	
9	1	F	17	OS	E	70	3	0	3	0	0	Yes	Yes	25	pT1	Pos	Pos	
10	2	F	11	OD	E	3	3	4					No			Pos	Pos	
11	2	M	11	OD	E	30	2	1					No			Pos	Pos	
12	1	F	25	OS	D	103	10	0	16	0	1	No	No			Pos	Pos	
13	1	M	23	OS	D	102	9	0	12	0	4	No	No			Pos	Pos	
14	1	M	22	OS	D	93	9	0	11	0	2	Yes	Yes	16	pt1	Pos	Pos	
15	2	M	15	OD	E	100	4	0					Yes	4	pT1	Pos	Pos	
16	1	M	29	OS	D	18	6	0	8	0	0	Yes	Yes	14	pT3d	Neg	Neg	18
17	1	M	7	OS	D	99	5	0	10	0	3	No	No			Pos	Pos	
18	2	M	6	OS	D	86	0	4					No			Pos	Pos	
19	1	M	19	OS	D	33	4	0	5	0	0	No	No			Pos	Pos	
20	1	M	20	OS	D	81	4	0	4	1	1	No	No			Pos	Pos	
21	1	M	38	OS	D	84	5	0	17	0	0	No	No			Pos	Pos	
22	2	M	12	OD	E	5	0	1					No			Pos	Pos	
23	2	F	26	OD	E	19	3	0					Yes	4	pT3b	Neg	Neg	19
24	2	F	12	OD	E	77	2	0					Yes	2	pT1	Pos	Pos	
25	2	M	11	OD	E	88	4	1					No			Pos	Pos	
26	2	F	4	OD	E	2	2	0					No			Pos	Pos	
27	1	M	15	OS	D	76	8	0	5	0	0	No	No			Pos	Pos	
28	2	F	6	OD	E	4	3	1					No			Pos	Pos	
29	2	M	9	OD	E	2	0	1					No			Pos	Pos	
30	2	M	35	OD	E	3	1	2					No			Pos	Pos	
31	1	F	27	OS	D	53	3	0	6	1	0	No	No			Pos	Pos	
32	2	F	22	OD	E	58	3	0					Yes	4	pT1	Pos	Pos	
33	2	F	9	OD	E	6	4	2					No			Pos	Pos	
34	1	M	2	OD	E	28	2	0	3	1	0	No	No			Neg	Pos	28
35	1	M	24	OS	C	82	4	0	10	0	3	No	No			Pos	Pos	
36	2	F	30	OD	E	83	1	0					Yes	1	pT3d	Pos	Pos	
37	1	M	23	OD	E	69	3	0	3	2	2	No	No			Pos	Pos	
38	1	F	35	OD	E	22	6	0	5	0	0	Yes	Yes	10	pT1	Neg	Neg	22
39	1	M	6	OD	E	66	2	0	2	0	2	No	No			Pos	Pos	
40	1	M	19	OS	C	64	3	0	3	0	0	No	No			Pos	Pos	
41	1	F	4	OS	C	58	2	0	3	0	0	No	No			Pos	Pos	
42	2	M	2	OD	E	78	2	1					Yes	11	pT1	Pos	Pos	
43	2	M	14	OS	B	71	4	3					No			Pos	Pos	
44	2	F	6	OD	E	78	2	0					Yes	14	pT2b	Pos	Pos	
45	1	M	42	OD	E	74	6	0	8	0	0	No	No			Pos	Pos	
46	2	M	18	OS	B	76	1	0					No			Pos	Pos	
47	2	F	11	OD	E	8	2	0					Yes	2	pT1	Neg	Neg	8
48	2	M	15	OD	E	82	3	0					Yes	3	pT1	Pos	Pos	
49	2	M	13	OD	E	67	4	0					Yes	4	pT1	Pos	Pos	
50	2	M	3	OS	B	58	0	0					No	0	pT3d	Pos	Pos	
51	1	M	4	OD	E	64	2	0	2	0	2	No	No			Pos	Pos	
52	1	M	28	OS	B	61	1	0	1	0	0	No	No			Pos	Pos	
53	2	M	10	OD	E	61	2	0					Yes	4	pT3b	Pos	Pos	
54	2	M	4	OD	E	55	3	0					Yes	13	N/A	Pos	Pos	
55	2	M	8	OD	E	66	2	3					Yes	10	pT1	Pos	Pos	
56	2	M	5	OS	B	55	5	0					No			Pos	Pos	
57	2	F	19	OS	A	83	0	0					No	0	pT1	Pos	Pos	
58	2	F	3	OD	E	87	2	0					Yes	11	pT1	Pos	Pos	
59	2	M	4	OD	E	18	3	0					Yes	3	pT1	Pos	Pos	
60	2	M	16	OD	E	18	10	2					Yes	17	pT4	Neg	Neg	18
61	2	M	23	OS	A	1	0	0					No	0	N/A	Pos	Pos	
62	1	M	4	OD	E	68	5	0	5	0	1	Yes	Yes	17	pT1	Pos	Pos	
63	2	M	8	OS	A	64	0	0					No	0	pT1	Pos	Pos	
64	2	M	10	OS	A	75	0	0					No	0	pT1	Pos	Pos	
65	1	M	2	OS	A	71	0	0	8	0	0	No	No			Pos	Pos	
66	1	F	19	OD	E	62	2	0	2	0	0	No	No			Pos	Pos	
67	3	M	38	OS	E	89	0	0					Yes	0	pT1	Pos	Pos	
68	3	F	28	OS	E	92	0	0					Yes	0	pT3b	Pos	Pos	
69	3	M	41	OD	E	3	0	0					Yes	0	pT1	Pos	Pos	
70	3	M	29	OS	E	88	0	0					Yes	0	pT3b	Pos	Pos	
71	3	F	18	OD	E	97	0	0					Yes	0	pT1	Pos	Pos	
72	3	M	27	OS	E	90	0	0					Yes	0	pT1	Pos	Pos	

Patient ID	Group	Sex	Age at Dx months	Studied Eye	IIRC	Follow-up Months	Pre-Tyl IVC #	Pre-Tyl IAC #	Dx to Tyl months	Post-Tyl IVC #	Post-Tyl Ivitc #	Post-Tyl Recur-rence	Enu	Dx to Enu	pTNM	OS	DDS	Dx to Death months
73	3	M	15	OD	E	92	0	0					Yes	0	pT3b	Pos	Pos	
74	3	F	28	OS	E	100	0	0					Yes	0	pt2a	Pos	Pos	
75	2	M	4	OD	E	87	2	0					Yes	33	pT1	Pos	Pos	
76	3	M	18	OD	E	86	0	0					Yes	0	pT3b	Pos	Pos	
77	3	M	2	OD	E	98	0	0					Yes	0	pT3b	Pos	Pos	
78	3	M	25	OS	E	100	0	0					Yes	0	pT1	Pos	Pos	
79	3	F	26	OD	E	9	0	0					Yes	0	pT3d	Neg	Neg	9
80	3	M	26	OD	E	95	0	0					Yes	0	pT1	Pos	Pos	
81	1	M	19	OD	E	94	5	0	15	2	1	No	No			Pos	Pos	
82	3	M	28	OS	E	8	0	0					Yes	0	pT3b	Pos	Pos	
83	3	M	21	OS	E	99	0	0					Yes	0	pT2b	Pos	Pos	
84	3	F	26	OD	E	0	0	0					Yes	0	pT3b	Pos	Pos	
85	3	M	36	OS	E	98	0	0					Yes	0	pT1	Pos	Pos	
86	3	M	15	OD	E	93	0	0					Yes	0	pT1	Pos	Pos	
87	3	M	20	OS	E	85	0	0					Yes	0	pT4	Pos	Pos	
88	2	M	29	OD	E	85	2	0					Yes	2	pT1	Pos	Pos	
89	3	F	60	OD	E	0	0	0					Yes	0	pT3b	Pos	Pos	
90	3	F	23	OD	E	84	0	0					Yes	0	pT3b	Pos	Pos	
91	3	M	50	OS	E	97	0	0					Yes	0	pT1	Pos	Pos	
92	3	F	15	OS	E	97	0	0					Yes	0	pT3b	Pos	Pos	
93	3	F	28	OD	E	86	0	0					Yes	0	pT4	Pos	Pos	
94	3	F	32	OD	E	86	0	0					Yes	0	pT1	Pos	Pos	
95	3	F	33	OS	E	4	0	0					Yes	0	pT3b	Pos	Pos	
96	2	F	33	OD	E	87	2	2					Yes	4	pT1	Pos	Pos	
97	2	M	6	OS	E	86	2	0					Yes	2	pT1	Pos	Pos	
98	2	F	39	OS	E	25	1	0					Yes	1	pT1	Pos	Pos	
99	3	M	31	OS	E	7	0	0					Yes	0	pT3b	Neg	Neg	7
100	3	F	20	OS	E	85	0	0					Yes	0	pT3b	Pos	Pos	
101	3	F	19	OS	E	3	0	0					Yes	0	pT1	Pos	Pos	
102	3	F	32	OD	E	82	0	0					Yes	0	pT3b	Pos	Pos	
103	3	F	21	OS	E	79	0	0					Yes	0	pT1	Pos	Pos	
104	3	M	29	OD	E	82	0	0					Yes	0	pT1	Pos	Pos	
105	3	F	12	OD	E	88	0	0					Yes	0	pt3b	Pos	Pos	
106	3	F	26	OS	E	54	0	0					Yes	0	pT3b	Pos	Pos	
107	3	F	4	OS	E	81	0	0					Yes	0	N/A	Pos	Pos	
108	3	M	15	OD	E	94	0	0					Yes	0	pT2a	Pos	Pos	
109	3	M	28	OD	E	9	0	0					Yes	0	pT4	Pos	Pos	
110	3	F	14	OS	E	96	0	0					Yes	0	pT3b	Pos	Pos	
111	3	M	23	OS	E	84	0	0					Yes	0	pT1	Pos	Pos	
112	3	M	55	OS	E	95	0	0					Yes	0	N/A	Pos	Pos	
113	2	M	4	OD	E	81	2	0					Yes	2	pt3b	Pos	Pos	
114	3	M	23	OD	E	11	0	0					Yes	0	pT4	Pos	Pos	
115	1	F	101	OS	E	95	1	0	4	0	6	Yes	Yes	10	pT2a	Pos	Pos	
116	1	F	55	OD	E	84	4	0	5	0	4	No	Yes	16	pT1	Pos	Pos	
117	3	F	47	OD	E	83	0	0					Yes	1	pT2b	Pos	Pos	
118	3	F	32	OD	E	17	0	0					Yes	0	pT4	Neg	Neg	17
119	2	M	31	OD	E	84	6	0					Yes	7	pT1	Pos	Pos	
120	2	M	20	OD	E	85	2	0					Yes	2	pT1	Pos	Pos	
121	3	M	11	OD	E	8	0	0					Yes	0	pT2a	Pos	Pos	
122	3	M	13	OD	E	94	0	0					Yes	0	pT3b	Pos	Pos	
123	3	M	15	OS	E	94	0	0					Yes	0	pT2b	Pos	Pos	
124	3	M	4	OD	E	86	0	0					Yes	0	N/A	Pos	Pos	
125	3	M	27	OS	E	83	0	0					Yes	0	pT1	Pos	Pos	
126	3	M	22	OD	E	36	0	0					Yes	0	pT1	Pos	Pos	
127	3	F	22	OS	E	79	0	0					Yes	1	pT1	Pos	Pos	
128	3	M	13	OS	E	92	0	0					Yes	0	pT3d	Pos	Pos	
129	3	M	30	OD	E	79	0	0					Yes	0	pT2a	Pos	Pos	
130	3	M	54	OS	E	92	0	0					Yes	0	pT2a	Pos	Pos	
131	3	M	5	OS	E	84	0	0					Yes	0	pT1	Pos	Pos	
132	3	M	14	OS	E	85	0	0					Yes	0	pT3a	Pos	Pos	
133	3	M	8	OD	E	82	0	0					Yes	0	pT1	Pos	Pos	
134	3	M	21	OS	E	81	0	0					Yes	0	pT2a	Pos	Pos	
135	3	M	22	OS	E	84	0	0					Yes	0	pT1	Pos	Pos	
136	3	M	61	OD	E	91	0	0					Yes	0	pT1	Pos	Pos	
137	3	F	36	OS	E	82	0	0					Yes	0	pT1	Pos	Pos	
138	1	M	5	OD	E	78	5	0	7	0	0	Yes	Yes	24	pT1	Pos	Pos	
139	3	F	20	OS	E	78	0	0					Yes	0	pT1	Pos	Pos	
140	3	M	10	OS	E	16	0	0					Yes	0	pT1	Pos	Pos	
141	3	F	5	OS	E	91	0	0					Yes	0	pT1	Pos	Pos	
142	1	F	7	OS	E	89	4	0	6	0	1	No	No			Pos	Pos	
143	3	M	20	OS	E	83	0	0					Yes	0	N/A	Pos	Pos	
144	3	F	11	OD	E	88	0	0					Yes	0	pT3b	Pos	Pos	

Safety of Tylectomy in Salvage of Eyes Affected by Retinoblastoma
Raw data, Table S2

Patient ID	Group	Sex	Age at Dx months	Studied Eye	IIRC	Follow-up Months	Pre-Tyl IVC #	Pre-Tyl IAC #	Dx to Tyl months	Post-Tyl IVC #	Post-Tyl Ivitc #	Post-Tyl Recur-rence	Enu	Dx to Enu	pTNM	OS	DDS	Dx to Death months
145	3	M	43	OD	E	74	0	0					Yes	0	pT3b	Pos	Pos	
146	3	M	34	OS	E	90	0	0					Yes	0	pT3b	Pos	Pos	
147	2	M	18	OD	E	19	2	0					Yes	10	pT4	Neg	Neg	19
148	3	M	16	OS	E	79	0	0					Yes	0	pT3b	Pos	Pos	
149	2	F	22	OS	E	73	1	0					Yes	1	pT1	Pos	Pos	
150	2	M	28	OD	E	88	2	0					Yes	2	pT1	Pos	Pos	
151	3	M	25	OD	E	13	0	0					Yes	0	pT1	Pos	Pos	
152	3	F	2	OD	E	82	0	0					Yes	0	N/A	Pos	Pos	
153	3	F	16	OS	E	0	0	0					Yes	0	pT1	Pos	Pos	
154	3	M	36	OS	E	5	0	0					Yes	0	pT3b	Neg	Neg	5
155	3	M	77	OS	E	17	0	0					Yes	1	N/A	Pos	Pos	
156	3	M	19	OS	E	22	0	0					Yes	0	pT1	Pos	Pos	
157	3	F	23	OD	E	89	0	0					Yes	0	pT1	Pos	Pos	
158	3	F	10	OS	E	16	0	0					Yes	0	pT3b	Pos	Pos	
159	3	F	23	OS	E	78	0	0					Yes	0	pT3b	Pos	Pos	
160	3	M	23	OD	E	81	0	0					Yes	0	pT1	Pos	Pos	
161	3	M	26	OS	E	72	0	0					Yes	0	pT3b	Pos	Pos	
162	3	F	33	OS	E	88	0	0					Yes	0	pT1	Pos	Pos	
163	1	F	20	OS	D	75	2	0	2	0	1	Yes	Yes	3	pT1	Pos	Pos	
164	3	F	45	OD	E	75	0	0					Yes	0	pT2a	Pos	Pos	
165	3	M	16	OS	E	86	0	0					Yes	0	pT3b	Pos	Pos	
166	2	M	49	OD	E	80	2	0					Yes	2	pT3b	Pos	Pos	
167	3	M	71	OS	E	81	0	0					Yes	0	pT1	Pos	Pos	
168	3	M	22	OS	E	74	0	0					Yes	0	pT2a	Pos	Pos	
169	3	M	3	OD	E	20	0	0					Yes	12	pT4	Neg	Neg	20
170	3	M	29	OS	E	10	0	0					Yes	0	pT3b	Neg	Neg	10
171	3	M	35	OD	E	27	0	0					Yes	0	pT4	Neg	Neg	27
172	1	F	25	OD	E	75	3	0	3	1	0	No	No			Pos	Pos	
173	3	F	17	OS	E	86	0	0					Yes	0	pT3b	Pos	Pos	
174	3	F	21	OS	E	87	0	0					Yes	0	pT2a	Pos	Pos	
175	1	F	100	OD	E	6	1	0	1	6	0	Yes	No			Neg	Pos	6
176	1	M	8	OD	E	77	3	0	3	1	1	No	No			Pos	Pos	
177	1	M	13	OD	E	76	3	0	3	0	0	No	No			Pos	Pos	
178	3	M	17	OD	E	73	0	0					Yes	0	pT3b	Pos	Pos	
179	3	F	2	OS	E	86	0	0					Yes	0	pT1	Pos	Pos	
180	1	M	104	OD	E	76	0	0	0	6	0	No	No			Pos	Pos	
181	3	M	111	OD	E	72	0	0					Yes	0	pT3b	Pos	Pos	
182	3	F	7	OS	E	75	0	0					Yes	0	pT1	Pos	Pos	
183	3	M	15	OS	E	75	0	0					Yes	1	pT1	Pos	Pos	
184	3	M	28	OS	E	13	0	0					Yes	0	N/A	Pos	Pos	
185	2	F	34	OS	E	85	2	0					Yes	2	pT1	Pos	Pos	
186	2	M	6	OD	E	10	1	0					No			Pos	Pos	
187	3	F	44	OD	E	85	0	0					Yes	0	pT2b	Pos	Pos	
188	1	M	20	OD	E	76	3	0	3	1	0	Yes	No			Pos	Pos	
189	1	M	40	OD	E	71	2	0	2	2	1	Yes	Yes	26	pT3a	Pos	Pos	
190	3	M	14	OS	E	76	0	0					Yes	0	pT3a	Pos	Pos	
191	2	F	30	OS	E	59	0	2					Yes	3	N/A	Pos	Pos	
192	2	M	4	OS	E	78	1	0					Yes	3	pT1	Pos	Pos	
193	1	F	15	OD	E	73	1	0	1	2	0	No	No			Pos	Pos	
194	3	F	55	OS	E	76	0	0					Yes	0	pT3a	Pos	Pos	
195	3	F	54	OS	E	35	0	0					Yes	0	pT1	Pos	Pos	
196	1	F	32	OD	E	6	3	0	3	1	0	No	No			Pos	Pos	
197	1	M	31	OS	E	74	0	0	1	3	0	Yes	Yes	11	pT1	Pos	Pos	
198	3	M	24	OD	E	10	0	0					Yes	0	pT3d	Neg	Neg	11
199	3	M	26	OD	E	47	0	0					Yes	0	pT3b	Pos	Pos	
200	1	F	7	OD	E	66	3	0	2	2	1	Yes	Yes	14	pT3a	Pos	Pos	
201	3	F	11	OD	E	77	0	0					Yes	0	pT1	Pos	Pos	
202	2	F	22	OS	E	69	2	0					Yes	3	pT2a	Pos	Pos	
203	2	F	22	OD	E	77	2	0					Yes	2	pT1	Pos	Pos	
204	3	F	56	OS	E	65	0	0					Yes	0	pT1	Pos	Pos	
205	2	M	37	OS	E	82	2	0					Yes	2	pT1	Pos	Pos	
206	2	M	51	OD	E	73	6	2					Yes	8	pT1	Pos	Pos	
207	1	M	22	OD	E	71	2	0	2	1	0	Yes	No			Pos	Pos	
208	3	F	14	OD	E	73	0	0					Yes	0	pT1	Pos	Pos	
209	2	F	3	OD	E	14	0	4					Yes	7	pT4	Neg	Neg	14
210	3	M	21	OD	E	67	0	0					Yes	0	pT3b	Pos	Pos	
211	1	F	27	OS	E	65	3	0	3	2	3	No	No			Pos	Pos	
212	3	F	31	OS	E	72	0	0					Yes	0	pT3b	Pos	Pos	
213	1	F	2	OS	E	63	3	0	3	1	0	No	No			Pos	Pos	
214	3	M	27	OD	E	80	0	0					Yes	0	N/A	Pos	Pos	
215	2	M	27	OS	E	72	2	0					Yes	2	pT1	Pos	Pos	
216	3	M	16	OS	E	73	0	0					Yes	0	pT1	Pos	Pos	

Safety of Tylectomy in Salvage of Eyes Affected by Retinoblastoma
Raw data, Table S2

Patient ID	Group	Sex	Age at Dx months	Studied Eye	IIRC	Follow-up Months	Pre-Tyl IVC #	Pre-Tyl IAC #	Dx to Tyl months	Post-Tyl IVC #	Post-Tyl Ivitc #	Post-Tyl Recur-rence	Enu	Dx to Enu	pTNM	OS	DDS	Dx to Death months
217	2	F	2	OD	E	73	2	0					Yes	2	pT1	Pos	Pos	
218	1	M	29	OD	E	64	1	0	0	1	3	Yes	Yes	5	pT1	Pos	Pos	
219	1	M	19	OD	E	66	5	0	6	0	0	Yes	No			Pos	Pos	
220	3	F	25	OD	E	67	0	0					Yes	0	pT1	Pos	Pos	
221	2	F	16	OS	E	2	2	0					Yes	2	pT1	Pos	Pos	
222	3	M	10	OS	E	71	0	0					Yes	0	pT2a	Pos	Pos	
223	2	F	3	OD	E	5	5	0					No			Pos	Pos	
224	1	M	19	OD	E	71	3	0	2	3	0	No	No			Pos	Pos	
225	1	F	35	OD	E	67	1	0	2	3	3	No	No			Pos	Pos	
226	2	M	57	OD	E	78	2	0					Yes	3	pt4	Pos	Pos	
227	3	M	17	OS	E	62	0	0					Yes	0	pT1	Pos	Pos	
228	1	M	20	OD	E	69	3	2	5	0	0	No	No			Pos	Pos	
229	3	M	30	OD	E	65	0	0					Yes	0	pT3b	Pos	Pos	
230	2	M	11	OD	E	77	2	0					Yes	4	pT1	Pos	Pos	
231	1	F	14	OD	E	69	1	0	1	2	1	No	No			Pos	Pos	
232	2	M	39	OS	E	62	2	0					Yes	3	pT1	Pos	Pos	
233	3	F	49	OS	E	77	0	0					Yes	0	pT3b	Pos	Pos	
234	3	M	18	OS	E	49	0	0					Yes	0	pT1	Pos	Pos	
235	3	M	28	OD	E	65	0	0					Yes	1	N/A	Pos	Pos	
236	3	F	29	OS	E	68	0	0					Yes	0	pT3b	Pos	Pos	
237	3	F	29	OD	E	72	0	0					Yes	0	pT1	Pos	Pos	
238	2	F	14	OD	E	1	2	0					No			Pos	Pos	
239	3	M	20	OS	E	63	0	0					Yes	0	pT1	Pos	Pos	
240	1	M	34	OD	E	64	2	0	3	1	1	No	No			Pos	Pos	
241	3	M	8	OD	E	68	0	0					Yes	0	N/A	Pos	Pos	
242	3	M	17	OD	E	65	0	0					Yes	0	pT3b	Pos	Pos	
243	2	F	40	OD	E	60	1	0					Yes	1	pT3b	Pos	Pos	
244	2	M	25	OS	E	62	0	1					Yes	1	pT3a	Pos	Pos	
245	1	M	70	OS	E	65	3	0	3	0	0	No	No			Pos	Pos	
246	2	M	9	OS	E	68	1	0					Yes	1	pT1	Pos	Pos	
247	3	F	22	OD	E	3	0	0					Yes	0	pT1	Pos	Pos	
248	3	F	11	OD	E	74	0	0					Yes	0	pT1	Pos	Pos	
249	3	F	19	OD	E	73	0	0					Yes	1	pT1	Pos	Pos	
250	3	F	20	OD	E	75	0	0					Yes	1	pT3b	Pos	Pos	
251	1	M	81	OS	E	68	0	0	1	1	0	No	Yes	2	pT1	Pos	Pos	
252	3	M	16	OD	E	64	0	0					Yes	0	pT1	Pos	Pos	
253	2	M	18	OS	E	60	2	0					Yes	2	pT3b	Pos	Pos	
254	2	M	27	OS	E	74	1	0					Yes	1	pT1	Pos	Pos	
255	2	M	20	OD	E	63	1	0					Yes	1	pT1	Pos	Pos	
256	2	F	17	OS	E	74	1	0					Yes	1	pT1	Pos	Pos	
257	1	M	42	OS	E	64	0	0	0	4	1	No	No			Pos	Pos	
258	2	F	40	OS	E	60	0	3					Yes	36	pT3d	Pos	Pos	
259	3	F	28	OD	E	4	0	0					Yes	0	pT4	Neg	Neg	4
260	3	F	11	OS	E	66	0	0					Yes	0	pT3b	Pos	Pos	
261	3	M	19	OD	E	66	0	0					Yes	0	pT3d	Pos	Pos	
262	3	M	18	OD	E	58	0	0					Yes	0	pT3d	Pos	Pos	
263	3	F	39	OD	E	8	0	0					Yes	0	pT3a	Pos	Pos	
264	3	M	12	OD	E	62	0	0					Yes	0	pT1	Pos	Pos	
265	1	M	6	OD	E	60	0	0	0	3	1	No	No			Pos	Pos	
266	3	M	23	OD	E	11	0	0					Yes	0	pT3b	Neg	Neg	11
267	3	M	5	OD	E	59	0	0					Yes	0	pT1	Pos	Pos	
268	3	F	21	OD	E	58	0	0					Yes	0	pT1	Pos	Pos	
269	3	M	34	OD	E	57	0	0					Yes	0	pT3b	Pos	Pos	
270	3	M	17	OS	E	71	0	0					Yes	0	pT1	Pos	Pos	
271	3	M	31	OS	E	61	0	0					Yes	0	pT3b	Pos	Pos	
272	3	F	18	OS	E	60	0	0					Yes	0	pT1	Pos	Pos	
273	3	M	1	OS	E	59	0	0					Yes	1	pT1	Pos	Pos	
274	1	M	34	OD	E	59	0	0	0	3	0	No	No			Pos	Pos	
275	3	M	28	OD	E	59	0	0					Yes	0	pT1	Pos	Pos	
276	3	F	24	OS	E	9	0	0					Yes	0	N/A	Neg	Neg	9
277	1	F	20	OD	E	64	2	0	2	0	0	No	No			Pos	Pos	
278	2	F	13	OD	E	59	2	1					Yes	3	pT1	Pos	Pos	
279	2	F	22	OD	E	62	1	0					Yes	1	N/A	Pos	Pos	
280	3	M	33	OD	E	59	0	0					Yes	0	pT2b	Pos	Pos	
281	3	F	19	OS	E	66	0	0					Yes	0	pT1	Pos	Pos	
282	3	M	18	OS	E	58	0	0					Yes	0	pT4	Pos	Pos	
283	2	F	25	OS	E	56	1	0					Yes	1	pT1	Pos	Pos	
284	2	M	23	OD	E	61	3	0					Yes	3	pT3a	Pos	Pos	
285	2	M	31	OD	E	66	1	0					Yes	1	pT1	Pos	Pos	
286	2	M	18	OD	E	58	3	0					Yes	3	pT1	Pos	Pos	
287	2	F	19	OS	E	61	1	0					Yes	1	pT1	Pos	Pos	
288	2	F	18	OD	E	61	0	3					No			Pos	Pos	

Safety of Tylectomy in Salvage of Eyes Affected by Retinoblastoma
Raw data, Table S2

Patient ID	Group	Sex	Age at Dx months	Studied Eye	IIRC	Follow-up Months	Pre-Tyl IVC #	Pre-Tyl IAC #	Dx to Tyl months	Post-Tyl IVC #	Post-Tyl Ivitc #	Post-Tyl Recurrence	Enu	Dx to Enu	pTNM	OS	DDS	Dx to Death months
289	2	M	46	OS	E	2	0	2					Yes	2	pT1	Pos	Pos	
290	2	F	38	OS	E	68	2	0					Yes	22	pT4	Pos	Pos	
291	3	M	31	OD	E	55	0	0					Yes	0	pT1	Pos	Pos	
292	2	M	18	OD	E	16	1	0					Yes	8	pT4	Neg	Neg	17
293	1	M	4	OD	E	60	3	0	4	1	0	No	No			Pos	Pos	
294	2	F	16	OS	E	52	4	2					Yes	7	N/A	Pos	Pos	
295	1	F	4	OD	E	62	0	1	1	1	0	No	No			Pos	Pos	
296	3	F	35	OD	E	60	0	0					Yes	0	pT1	Pos	Pos	
297	2	M	22	OD	E	12	1	0					Yes	1	pT3b	Neg	Neg	12
298	2	M	53	OS	E	53	1	0					Yes	2	pT1	Pos	Pos	
299	2	F	20	OD	E	68	1	0					Yes	1	pT3a	Pos	Pos	
300	3	M	24	OD	E	54	0	0					Yes	0	pT3b	Pos	Pos	
301	2	M	23	OD	E	67	2	0					Yes	2	pT1	Pos	Pos	
302	3	M	18	OD	E	66	0	0					Yes	0	pT3d	Pos	Pos	
303	3	F	32	OD	E	0	0	0					Yes	0	pT1	Pos	Pos	
304	2	M	25	OD	E	53	0	2					Yes	2	pT1	Pos	Pos	
305	2	F	27	OS	E	51	1	0					Yes	1	pT1	Pos	Pos	
306	2	M	2	OD	E	56	4	0					Yes	15	pT1	Pos	Pos	
307	2	M	20	OS	E	67	2	0					Yes	2	pT1	Pos	Pos	
308	2	M	1	OS	E	55	1	0					Yes	3	pT1	Pos	Pos	
309	1	M	34	OD	E	65	2	0	3	2	0	Yes	Yes	12	pT2b	Pos	Pos	
310	2	F	16	OD	D	88	5	0					No			Pos	Pos	
311	1	F	5	OD	D	83	8	0	12	5	0	No	No			Pos	Pos	
312	2	M	5	OD	D	82	5	0					No			Pos	Pos	
313	1	F	15	OD	D	90	4	2	9	0	4	No	No			Pos	Pos	
314	2	M	5	OD	D	38	2	0					No			Neg	Neg	38
315	2	F	6	OD	D	8	2	3					No			Pos	Pos	
316	2	F	8	OD	D	27	2	0					No			Neg	Neg	27
317	2	F	20	OD	D	78	4	7					No			Pos	Pos	
318	2	M	20	OD	D	42	3	0					Yes	26	N/A	Pos	Pos	
319	2	M	17	OD	D	4	4	0					No			Pos	Pos	
320	1	M	17	OS	E	69	4	9	50	2	0	Yes	Yes	58	pT3d	Pos	Pos	
321	2	F	15	OD	D	5	4	0					No			Pos	Pos	
322	2	M	14	OD	D	14	2	0					No			Pos	Pos	
323	2	F	7	OD	D	61	2	0					Yes	46	N/A	Pos	Pos	
324	1	M	3	OD	D	67	3	1	21	2	0	No	No			Pos	Pos	
325	2	F	8	OS	E	15	1	0					No			Neg	Neg	15
326	2	F	15	OD	D	23	6	0					No			Neg	Neg	23
327	1	M	7	OS	D	23	8	0	15	0	0	Yes	No			Neg	Neg	23
328	2	M	13	OD	D	94	8	0					Yes	10	pT2a	Pos	Pos	
329	1	F	4	OS	D	88	7	0	13	0	0	Yes	No			Pos	Pos	
330	1	M	5	OD	D	85	7	2	9	0	0	Yes	No			Pos	Pos	
331	1	M	18	OD	D	87	6	0	12	0	0	No	No			Pos	Pos	
332	1	M	22	OD	D	88	3	3	9	1	0	Yes	No			Pos	Pos	
333	1	M	17	OD	D	77	5	2	10	0	0	No	No			Pos	Pos	
334	1	F	7	OS	D	25	6	0	11	0	0	Yes	No			Neg	Neg	25
335	1	M	3	OD	D	18	3	0	6	0	0	No	No			Pos	Pos	
336	1	M	12	OD	D	80	8	0	7	0	0	No	No			Pos	Pos	
337	1	F	26	OS	D	29	6	0	8	0	0	Yes	No			Neg	Neg	29
338	2	F	6	OD	D	41	3	0					No			Neg	Neg	41
339	1	M	27	OD	D	76	5	0	8	0	0	No	No			Pos	Pos	
340	2	M	10	OD	D	86	0	4					No			Pos	Pos	
341	2	M	9	OD	D	6	6	0					Yes	6	N/A	Pos	Pos	
342	2	M	19	OD	D	2	2	0					No			Pos	Pos	
343	2	M	11	OD	D	2	2	0					No			Pos	Pos	
344	1	M	27	OD	D	73	5	0	6	0	0	Yes	Yes	41	pT2b	Pos	Pos	
345	2	F	9	OD	D	2	3	0					No			Pos	Pos	
346	1	M	8	OD	D	76	4	0	4	0	0	Yes	No			Pos	Pos	
347	1	M	13	OD	D	75	4	0	4	0	0	Yes	No			Pos	Pos	
348	2	M	8	OD	D	3	1	1					No			Pos	Pos	
349	1	F	14	OD	D	73	3	0	3	0	0	No	No			Pos	Pos	
350	2	F	7	OD	D	18	6	0					No			Pos	Pos	
351	1	M	3	OD	D	78	3	0	3	1	2	No	No			Pos	Pos	
352	2	M	19	OD	D	4	5	0					No			Pos	Pos	
353	2	M	25	OD	D	79	6	3					Yes	28	N/A	Pos	Pos	
354	2	F	13	OD	D	78	4	0					No			Pos	Pos	
355	2	M	22	OD	D	63	5	1					No			Pos	Pos	
356	2	M	1	OD	D	3	3	0					No			Pos	Pos	
357	2	M	10	OD	D	20	2	0					No			Neg	Neg	20
358	2	F	13	OD	D	3	3	0					No			Pos	Pos	
359	2	F	12	OD	D	76	6	0					No			Pos	Pos	
360	2	F	24	OD	D	62	4	0					Yes	7	pT2b	Pos	Pos	

Safety of Tylectomy in Salvage of Eyes Affected by Retinoblastoma
Raw data, Table S2

Patient ID	Group	Sex	Age at Dx months	Studied Eye	IIRC	Follow-up Months	Pre-Tyl IVC #	Pre-Tyl IAC #	Dx to Tyl months	Post-Tyl IVC #	Post-Tyl Ivitc #	Post-Tyl Recur-rence	Enu	Dx to Enu	pTNM	OS	DDS	Dx to Death months
361	2	M	19	OD	D	2	2	0					No			Pos	Pos	
362	2	F	4	OD	D	69	6	0					No			Pos	Pos	
363	2	M	25	OD	D	10	3	1					No			Neg	Neg	10
364	1	M	16	OD	D	67	3	0	3	0	0	No	No			Pos	Pos	
365	2	F	6	OD	D	8	6	0					No			Pos	Pos	
366	2	M	20	OS	D	8	4	0					No			Neg	Neg	8
367	1	F	17	OD	D	64	5	1	5	0	0	No	No			Pos	Pos	
368	2	M	3	OD	D	4	4	0					No			Pos	Pos	
369	2	F	14	OD	D	33	9	0					No			Pos	Pos	
370	2	M	9	OD	D	2	2	0					No			Pos	Pos	
371	2	F	5	OD	D	62	1	3					Yes	19	N/A	Pos	Pos	
372	1	F	18	OD	D	63	5	0	5	0	0	No	No			Pos	Pos	
373	1	M	25	OS	D	67	5	0	13	7	3	No	No			Pos	Pos	
374	2	F	13	OD	D	2	2	0					No			Pos	Pos	
375	1	F	2	OD	D	82	4	3	18	1	1	Yes	Yes	34	pT3a	Pos	Pos	
376	1	M	11	OD	D	68	2	0	3	0	0	No	No			Pos	Pos	
377	1	M	23	OD	D	64	3	0	2	0	0	No	No			Pos	Pos	
378	2	F	4	OD	D	30	1	0					No			Neg	Neg	30
379	1	F	13	OD	D	56	5	0	5	0	0	No	No			Pos	Pos	
380	1	F	35	OD	D	60	1	1	2	2	0	Yes	Yes	10	pT3b	Pos	Pos	
381	2	F	2	OD	D	23	2	0					No			Neg	Neg	23
382	2	M	1	OD	D	101	8	0					Yes	9	pT1	Pos	Pos	
383	1	M	8	OD	D	90	6	0	12	0	0	Yes	Yes	23	pT1	Pos	Pos	
384	2	F	4	OD	D	81	3	1					No			Pos	Pos	
385	2	F	3	OD	D	8	4	0					No			Pos	Pos	
386	2	M	6	OD	D	93	6	0					Yes	12	pT1	Pos	Pos	
387	2	M	31	OD	D	97	2	0					Yes	1	pT1	Pos	Pos	
388	1	M	14	OD	D	78	6	2	24	0	0	Yes	Yes	25	N/A	Pos	Pos	
389	2	F	2	OD	D	82	5	0					Yes	41	pT3a	Pos	Pos	
390	1	M	5	OD	D	76	6	0	9	0	4	Yes	No			Pos	Pos	
391	1	M	18	OD	D	87	2	3	6	0	1	No	No			Pos	Pos	
392	1	M	4	OD	D	79	4	3	7	0	0	No	No			Pos	Pos	
393	1	M	3	OD	D	73	6	0	8	0	2	No	No			Pos	Pos	
394	1	M	5	OD	D	74	6	0	6	0	0	Yes	Yes	50	pT1	Pos	Pos	
395	1	M	7	OD	D	67	3	0	3	0	0	No	No			Pos	Pos	
396	1	F	8	OD	D	79	3	0	3	0	0	Yes	No			Pos	Pos	
397	2	F	13	OD	D	65	3	0					Yes	3	pT1	Pos	Pos	
398	1	F	22	OS	B	63	3	0	3	0	0	No	No			Pos	Pos	
399	2	F	14	OD	D	64	3	0					Yes	3	pT1	Pos	Pos	
400	1	F	15	OD	D	2	2	0	2	0	0	No	No			Pos	Pos	
401	2	M	3	OD	D	60	9	0					No			Pos	Pos	
402	2	M	4	OD	D	2	2	0					No			Pos	Pos	
403	2	M	4	OD	D	61	3	3					No			Pos	Pos	
404	2	F	31	OS	B	57	3	0					No			Pos	Pos	
405	2	F	30	OS	A	93	0	0					No	0	pT1	Pos	Pos	
406	2	F	15	OD	D	97	5	0					Yes	12	pT1	Pos	Pos	
407	2	F	7	OS	A	82	0	0					No	0	pT1	Pos	Pos	
408	1	M	12	OD	D	70	2	0	3	0	0	No	No			Pos	Pos	
409	2	F	48	OD	D	75	3	0					Yes	3	pT1	Pos	Pos	
410	2	M	16	OD	D	9	5	3					No			Pos	Pos	
411	2	F	44	OS	A	54	0	0					No	0	N/A	Pos	Pos	
412	2	M	16	OD	D	12	12	0					No			Pos	Pos	
413	2	F	5	OD	D	62	4	1					No			Pos	Pos	
414	2	F	12	OD	D	11	1	2					No			Pos	Pos	
415	3	F	39	OD	D	102	0	0					Yes	0	pT1	Pos	Pos	
416	2	F	21	OS	D	100	8	2					Yes	11	pT1	Pos	Pos	
417	3	M	13	OS	D	102	0	0					Yes	1	pT1	Pos	Pos	
418	1	M	25	OS	D	100	7	0	16	0	0	No	No			Pos	Pos	
419	2	M	13	OS	D	91	4	1					No			Pos	Pos	
420	3	F	28	OD	D	90	0	0					Yes	0	pT1	Pos	Pos	
421	3	M	34	OD	D	96	0	0					Yes	0	pT1	Pos	Pos	
422	3	M	12	OD	D	67	0	0					Yes	0	pT1	Pos	Pos	
423	2	F	17	OS	D	86	2	3					No			Pos	Pos	
424	2	M	22	OD	D	6	1	3					No			Pos	Pos	
425	1	M	40	OS	D	91	0	3	19	0	2	Yes	No			Pos	Pos	
426	2	M	32	OD	D	94	5	1					No			Pos	Pos	
427	3	M	25	OD	D	87	0	0					Yes	0	pT1	Pos	Pos	
428	3	M	32	OD	D	95	0	0					Yes	0	pT1	Pos	Pos	
429	2	M	34	OD	D	100	3	0					Yes	3	pT1	Pos	Pos	
430	2	F	25	OD	D	95	4	0					Yes	4	pT1	Pos	Pos	
431	3	F	37	OD	D	98	0	0					Yes	0	pT1	Pos	Pos	
432	3	F	31	OD	D	0	0	0					Yes	0	pT1	Pos	Pos	

Safety of Tylectomy in Salvage of Eyes Affected by Retinoblastoma
Raw data, Table S2

Patient ID	Group	Sex	Age at Dx months	Studied Eye	IIRC	Follow-up Months	Pre-Tyl IVC #	Pre-Tyl IAC #	Dx to Tyl months	Post-Tyl IVC #	Post-Tyl Ivitc #	Post-Tyl Recur-rence	Enu	Dx to Enu	pTNM	OS	DDS	Dx to Death months
433	3	M	54	OS	D	87	0	0					Yes	0	pT1	Pos	Pos	
434	2	F	40	OS	D	100	3	0					No			Pos	Pos	
435	2	F	3	OS	D	100	2	2					No			Pos	Pos	
436	3	M	34	OD	D	100	0	0					Yes	0	pT1	Pos	Pos	
437	3	M	20	OS	D	89	0	0					Yes	0	pT1	Pos	Pos	
438	2	F	19	OD	D	92	1	0					Yes	0	pT1	Pos	Pos	
439	2	F	9	OD	D	94	6	0					Yes	11	pT1	Pos	Pos	
440	3	M	18	OD	D	89	0	0					Yes	0	pT1	Pos	Pos	
441	3	M	45	OS	D	99	0	0					Yes	0	pT1	Pos	Pos	
442	3	F	12	OS	D	99	0	0					Yes	0	pT3a	Pos	Pos	
443	2	M	3	OD	D	86	3	0					Yes	3	pT1	Pos	Pos	
444	2	F	39	OD	D	86	6	0					Yes	7	pT1	Pos	Pos	
445	3	F	6	OS	D	86	0	0					Yes	0	pT2a	Pos	Pos	
446	3	M	12	OD	D	99	0	0					Yes	0	pT1	Pos	Pos	
447	1	F	29	OS	D	94	3	2	7	0	0	No	No			Pos	Pos	
448	3	M	18	OD	D	94	0	0					Yes	0	pT1	Pos	Pos	
449	3	F	7	OS	D	84	0	0					Yes	0	pT1	Pos	Pos	
450	2	M	34	OD	D	96	4	0					Yes	4	pT1	Pos	Pos	
451	1	F	24	OD	D	93	4	1	9	0	3	No	No			Pos	Pos	
452	3	M	65	OS	D	85	0	0					Yes	0	pT3b	Pos	Pos	
453	1	M	21	OS	D	66	0	3	14	0	1	Yes	Yes	25	N/A	Pos	Pos	
454	2	M	32	OS	D	11	2	2					Yes	9	pT4	Neg	Neg	11
455	2	M	27	OS	D	98	6	0					Yes	6	pT1	Pos	Pos	
456	2	F	8	OS	D	87	4	0					No			Pos	Pos	
457	2	F	44	OD	D	98	4	0					Yes	9	pT1	Pos	Pos	
458	2	M	4	OS	D	60	5	0					Yes	10	pT3a	Pos	Pos	
459	2	M	22	OD	D	84	1	0					Yes	1	pT3b	Pos	Pos	
460	2	F	46	OD	D	94	2	0					Yes	2	pT1	Pos	Pos	
461	2	M	2	OD	D	81	0	2					No			Pos	Pos	
462	2	M	22	OS	D	98	2	0					Yes	2	pT1	Pos	Pos	
463	2	M	23	OS	D	75	5	0					Yes	5	pT1	Pos	Pos	
464	3	F	14	OD	D	81	0	0					Yes	0	pT1	Pos	Pos	
465	3	F	26	OD	D	97	0	0					Yes	0	pT1	Pos	Pos	
466	2	F	28	OS	D	89	1	0					Yes	1	pT1	Pos	Pos	
467	2	M	20	OS	D	87	2	0					Yes	3	pT1	Pos	Pos	
468	3	M	22	OD	D	92	0	0					Yes	0	pT1	Pos	Pos	
469	2	F	27	OD	D	92	6	0					Yes	5	pT1	Pos	Pos	
470	2	F	43	OD	D	97	6	2					Yes	9	pT1	Pos	Pos	
471	3	M	30	OS	D	95	0	0					Yes	0	pT1	Pos	Pos	
472	2	M	35	OD	D	86	6	0					Yes	6	pT2b	Pos	Pos	
473	1	M	96	OS	D	85	6	2	8	4	0	Yes	Yes	22	pT1	Pos	Pos	
474	2	M	3	OS	D	82	3	0					Yes	4	pT1	Pos	Pos	
475	2	M	26	OD	D	86	1	0					Yes	1	pT1	Pos	Pos	
476	1	F	36	OS	D	85	6	0	10	0	1	No	No			Pos	Pos	
477	3	F	43	OS	D	96	0	0					Yes	0	pT1	Pos	Pos	
478	3	F	5	OD	D	90	0	0					Yes	0	pT1	Pos	Pos	
479	2	F	2	OD	D	82	5	0					Yes	4	pT1	Pos	Pos	
480	1	F	73	OS	D	90	3	0	4	0	0	No	No			Pos	Pos	
481	2	M	17	OS	D	82	4	0					Yes	4	pT1	Pos	Pos	
482	1	F	6	OD	D	84	6	0	13	0	0	No	No			Pos	Pos	
483	1	M	68	OS	D	95	2	0	5	0	4	No	No			Pos	Pos	
484	2	M	16	OS	D	82	4	0					Yes	8	pT1	Pos	Pos	
485	2	M	23	OD	D	16	1	0					Yes	1	pT1	Pos	Pos	
486	2	M	21	OS	D	88	3	1					Yes	5	pT1	Pos	Pos	
487	2	F	2	OD	D	15	0	2					Yes	15	N/A	Pos	Pos	
488	2	F	32	OS	D	52	0	4					No			Pos	Pos	
489	1	F	4	OD	D	92	3	2	9	0	0	Yes	No			Pos	Pos	
490	2	M	21	OD	D	93	2	0					Yes	2	pT1	Pos	Pos	
491	1	M	20	OS	D	84	5	0	6	0	2	No	No			Pos	Pos	
492	2	F	22	OS	D	5	5	0					Yes	5	N/A	Pos	Pos	
493	3	M	22	OD	D	83	0	0					Yes	1	pT1	Pos	Pos	
494	3	F	25	OD	D	94	0	0					Yes	0	pt1	Pos	Pos	
495	3	M	10	OD	D	77	0	0					Yes	1	pT3c	Pos	Pos	
496	2	M	45	OS	D	16	7	0					Yes	9	N/A	Pos	Pos	
497	1	F	4	OS	D	87	0	2	8	0	0	No	No			Pos	Pos	
498	3	M	32	OD	D	78	0	0					Yes	0	pT1	Pos	Pos	
499	3	F	23	OD	D	88	0	0					Yes	0	pT1	Pos	Pos	
500	3	F	21	OD	D	70	0	0					Yes	0	pT1	Pos	Pos	
501	3	M	33	OS	D	93	0	0					Yes	0	pT1	Pos	Pos	
502	2	M	22	OD	D	88	2	0					Yes	2	pT1	Pos	Pos	
503	1	F	8	OS	D	83	4	0	8	0	1	No	No			Pos	Pos	
504	3	F	25	OD	D	59	0	0					Yes	0	N/A	Pos	Pos	

Safety of Tylectomy in Salvage of Eyes Affected by Retinoblastoma
Raw data, Table S2

Patient ID	Group	Sex	Age at Dx months	Studied Eye	IIRC	Follow-up Months	Pre-Tyl IVC #	Pre-Tyl IAC #	Dx to Tyl months	Post-Tyl IVC #	Post-Tyl Ivitc #	Post-Tyl Recur-rence	Enu	Dx to Enu	pTNM	OS	DDS	Dx to Death months
505	1	F	24	OD	D	85	6	0	5	1	2	No	No			Pos	Pos	
506	2	F	22	OD	D	2	3	0					No			Pos	Pos	
507	3	F	13	OD	D	93	0	0					Yes	0	pT1	Pos	Pos	
508	2	F	20	OS	D	9	1	0					Yes	1	pT1	Pos	Pos	
509	3	M	7	OD	D	79	0	0					Yes	0	N/A	Pos	Pos	
510	3	F	22	OS	D	82	0	0					Yes	0	pT1	Pos	Pos	
511	2	F	32	OS	D	82	1	0					Yes	1	pT1	Pos	Pos	
512	3	M	9	OD	D	92	0	0					Yes	0	pT1	Pos	Pos	
513	3	M	23	OS	D	81	0	0					Yes	0	pT2a	Pos	Pos	
514	2	F	30	OS	D	81	2	0					Yes	4	pT1	Pos	Pos	
515	1	M	11	OD	D	76	6	0	6	0	0	Yes	No			Pos	Pos	
516	1	M	5	OD	D	76	6	0	8	0	0	Yes	Yes	16	pT4	Pos	Pos	
517	3	M	24	OS	D	84	0	0					Yes	0	pT1	Pos	Pos	
518	3	F	24	OS	D	92	0	0					Yes	0	pT1	Pos	Pos	
519	3	M	35	OD	D	76	0	0					Yes	0	pT1	Pos	Pos	
520	3	M	18	OD	D	92	0	0					Yes	0	pT1	Pos	Pos	
521	3	M	24	OS	D	79	0	0					Yes	0	pT1	Pos	Pos	
522	3	F	42	OD	D	81	0	0					Yes	0	pT1	Pos	Pos	
523	3	F	11	OS	D	36	0	0					Yes	0	pT1	Pos	Pos	
524	2	M	3	OD	D	92	4	0					Yes	4	pT2a	Pos	Pos	
525	2	M	21	OD	D	80	2	0					Yes	3	pT1	Pos	Pos	
526	3	M	89	OD	D	84	0	0					Yes	0	pT1	Pos	Pos	
527	2	M	21	OS	D	91	1	0					Yes	1	pT1	Pos	Pos	
528	2	F	7	OD	D	91	2	3					No			Pos	Pos	
529	2	M	2	OS	D	90	5	0					Yes	5	pT2b	Pos	Pos	
530	1	F	47	OS	D	74	0	4	9	0	1	No	No			Pos	Pos	
531	2	F	15	OS	D	7	2	2					No			Pos	Pos	
532	2	F	10	OD	D	83	1	0					Yes	1	pT1	Pos	Pos	
533	3	F	5	OD	D	85	0	0					Yes	0	N/A	Pos	Pos	
534	1	M	7	OS	D	80	3	0	6	0	0	No	No			Pos	Pos	
535	1	M	5	OS	D	77	4	0	6	0	1	No	No			Pos	Pos	
536	3	M	35	OD	D	90	0	0					Yes	0	pT1	Pos	Pos	
537	1	F	23	OS	D	37	1	3	7	0	0	Yes	No			Neg	Neg	37
538	2	F	17	OD	D	19	3	0					Yes	3	pT1	Pos	Pos	
539	1	F	6	OD	D	78	5	0	6	0	1	Yes	No			Pos	Pos	
540	2	F	17	OD	D	16	4	0					Yes	14	N/A	Pos	Pos	
541	3	F	22	OD	D	90	0	0					Yes	0	pT1	Pos	Pos	
542	2	M	33	OS	D	56	1	0					Yes	1	pT1	Pos	Pos	
543	2	M	3	OD	D	90	2	0					Yes	2	pT1	Pos	Pos	
544	2	F	31	OD	D	7	4	3					No			Pos	Pos	
545	1	M	29	OD	D	81	2	0	2	0	0	No	No			Pos	Pos	
546	1	F	12	OS	D	80	4	0	5	0	1	Yes	No			Pos	Pos	
547	3	M	35	OD	D	76	0	0					Yes	0	pT1	Pos	Pos	
548	1	M	18	OS	D	76	6	0	6	0	1	No	No			Pos	Pos	
549	3	M	27	OS	D	52	0	0					Yes	0	pT1	Pos	Pos	
550	3	M	30	OS	D	89	0	0					Yes	0	pT1	Pos	Pos	
551	3	M	11	OD	D	84	0	0					Yes	0	pT1	Pos	Pos	
552	3	M	28	OS	D	90	0	0					Yes	0	pT3a	Pos	Pos	
553	3	F	29	OS	D	89	0	0					Yes	0	pT1	Pos	Pos	
554	2	M	23	OS	D	72	1	0					Yes	1	pT1	Pos	Pos	
555	3	M	22	OD	D	82	0	0					Yes	0	pT1	Pos	Pos	
556	3	F	34	OD	D	81	0	0					Yes	0	pT3b	Pos	Pos	
557	2	F	23	OS	D	75	3	0					Yes	3	pT1	Pos	Pos	
558	3	M	25	OD	D	7	0	0					Yes	0	pT3b	Pos	Pos	
559	1	F	38	OD	D	68	4	0	3	0	1	No	No			Pos	Pos	
560	1	F	28	OD	D	77	4	0	4	0	0	Yes	Yes	10	pT3d	Neg	Pos	77
561	1	M	5	OD	D	77	6	0	6	0	1	Yes	Yes	27	pT1	Pos	Pos	
562	1	F	4	OS	D	76	1	2	6	0	0	No	No			Pos	Pos	
563	3	F	27	OS	D	88	0	0					Yes	0	pT1	Pos	Pos	
564	1	F	24	OS	D	79	3	0	3	1	0	No	No			Pos	Pos	
565	1	M	50	OS	D	76	1	3	5	0	1	No	No			Pos	Pos	
566	3	F	35	OS	D	88	0	0					Yes	1	pT1	Pos	Pos	
567	1	M	44	OD	D	80	6	0	6	1	2	Yes	Yes	22	pT2a	Pos	Pos	
568	1	M	27	OS	D	71	3	0	2	1	1	No	No			Pos	Pos	
569	1	F	7	OD	D	78	3	0	3	1	0	No	No			Pos	Pos	
570	3	F	20	OS	D	76	0	0					Yes	0	N/A	Pos	Pos	
571	1	M	29	OD	D	76	3	0	7	0	0	Yes	No			Pos	Pos	
572	1	F	13	OS	D	75	5	0	5	0	0	No	No			Pos	Pos	
573	1	M	19	OD	D	71	4	0	4	0	0	No	No			Pos	Pos	
574	3	M	3	OD	D	81	0	0					Yes	0	pT1	Pos	Pos	
575	1	M	34	OS	D	74	2	0	4	0	1	Yes	Yes	39	pT3c	Pos	Pos	
576	3	F	43	OD	D	65	0	0					Yes	0	pT1	Pos	Pos	

Safety of Tylectomy in Salvage of Eyes Affected by Retinoblastoma
Raw data, Table S2

Patient ID	Group	Sex	Age at Dx months	Studied Eye	IIRC	Follow-up Months	Pre-Tyl IVC #	Pre-Tyl IAC #	Dx to Tyl months	Post-Tyl IVC #	Post-Tyl Ivitc #	Post-Tyl Recur-rence	Enu	Dx to Enu	pTNM	OS	DDS	Dx to Death months
577	2	M	12	OS	D	87	2	0					Yes	3	pT1	Pos	Pos	
578	1	M	21	OD	D	74	3	0	2	0	0	Yes	Yes	9	pT2a	Pos	Pos	
579	2	F	31	OS	D	15	3	0					No			Pos	Pos	
580	1	F	8	OS	D	75	3	0	2	0	1	No	No			Pos	Pos	
581	2	F	12	OD	D	49	0	5					No			Pos	Pos	
582	1	F	30	OD	D	73	3	0	2	0	0	No	Yes	3	pT1	Pos	Pos	
583	1	M	13	OD	D	74	2	2	5	0	1	No	No			Pos	Pos	
584	2	M	15	OD	D	2	2	0					No			Pos	Pos	
585	1	F	4	OD	D	78	6	0	5	1	1	No	No			Pos	Pos	
586	2	M	13	OD	D	17	3	0					Yes	3	pT1	Pos	Pos	
587	1	M	28	OS	D	73	3	0	4	0	1	No	No			Pos	Pos	
588	2	F	24	OD	D	74	6	0					No			Pos	Pos	
589	3	M	53	OS	D	77	0	0					Yes	0	pT1	Pos	Pos	
590	2	F	27	OD	D	62	2	0					Yes	2	pT1	Pos	Pos	
591	1	M	9	OD	D	75	2	0	3	0	0	No	No			Pos	Pos	
592	3	M	21	OD	D	85	0	0					Yes	0	pT1	Pos	Pos	
593	1	F	8	OD	D	77	3	0	3	0	1	No	No			Pos	Pos	
594	2	M	36	OS	D	8	1	0					No			Pos	Pos	
595	3	F	39	OD	D	84	0	0					Yes	1	pT1	Pos	Pos	
596	2	M	21	OD	D	70	6	0					Yes	13	N/A	Pos	Pos	
597	1	F	56	OS	D	74	2	0	2	0	1	No	No			Pos	Pos	
598	3	M	33	OD	D	71	0	0					Yes	0	pT2a	Pos	Pos	
599	1	M	34	OD	D	79	2	0	2	2	1	No	No			Pos	Pos	
600	1	F	37	OD	D	71	3	0	3	0	0	Yes	Yes	36	pT2a	Pos	Pos	
601	3	F	12	OD	D	70	0	0					Yes	0	N/A	Pos	Pos	
602	1	M	22	OS	D	78	2	0	2	2	1	No	No			Pos	Pos	
603	1	M	36	OS	D	75	2	0	3	0	1	Yes	Yes	23	pT2a	Pos	Pos	
604	3	M	3	OS	D	75	0	0					Yes	3	pT1	Pos	Pos	
605	1	M	31	OD	D	75	4	0	3	1	1	Yes	Yes	6	pT4	Pos	Pos	
606	3	F	13	OD	D	50	0	0					Yes	0	pT3b	Pos	Pos	
607	1	F	28	OS	D	67	3	0	3	0	0	Yes	Yes	10	pT3a	Pos	Pos	
608	2	F	3	OD	D	48	6	0					No			Pos	Pos	
609	2	F	119	OS	D	3	1	1					No			Pos	Pos	
610	3	F	17	OD	D	69	0	0					Yes	1	pT1	Pos	Pos	
611	3	M	23	OD	D	71	0	0					Yes	3	pT1	Pos	Pos	
612	1	F	37	OD	D	73	4	1	5	0	0	No	No			Pos	Pos	
613	1	M	25	OD	D	69	0	2	2	1	3	No	No			Pos	Pos	
614	3	M	15	OS	D	29	0	0					Yes	19	pT4	Neg	Neg	29
615	2	M	20	OD	D	82	3	0					Yes	2	pT1	Pos	Pos	
616	2	M	30	OS	D	82	2	0					Yes	2	pT1	Pos	Pos	
617	2	M	23	OD	D	80	2	0					Yes	2	pT1	Pos	Pos	
618	1	M	13	OD	D	66	2	0	2	1	1	No	No			Pos	Pos	
619	3	F	25	OD	D	82	0	0					Yes	0	N/A	Pos	Pos	
620	2	F	4	OS	D	81	7	0					Yes	6	pT1	Pos	Pos	
621	1	M	34	OD	D	66	2	0	2	1	2	No	No			Pos	Pos	
622	1	F	31	OD	D	76	4	0	4	0	0	No	No			Pos	Pos	
623	2	M	20	OS	D	76	2	0					Yes	2	pT1	Pos	Pos	
624	2	F	44	OS	D	7	3	0					Yes	3	pT1	Pos	Pos	
625	1	M	22	OD	D	67	2	0	2	2	0	No	No			Pos	Pos	
626	3	F	30	OS	D	81	0	0					Yes	0	N/A	Pos	Pos	
627	2	M	17	OD	D	38	0	3					No			Pos	Pos	
628	1	M	48	OS	D	70	1	0	1	1	1	No	No			Pos	Pos	
629	1	F	15	OS	D	70	3	0	3	0	1	No	No			Pos	Pos	
630	1	F	14	OS	D	67	0	2	1	0	2	No	No			Pos	Pos	
631	1	F	23	OS	D	68	3	0	3	1	1	No	No			Pos	Pos	
632	1	F	30	OS	D	69	2	0	6	0	0	No	No			Pos	Pos	
633	1	M	29	OS	D	67	2	0	3	0	1	Yes	Yes	11	pT3c	Pos	Pos	
634	1	M	22	OD	D	69	0	0	0	2	3	No	No			Pos	Pos	
635	1	F	10	OD	D	68	1	0	1	0	0	No	No			Pos	Pos	
636	3	M	37	OS	D	69	0	0					Yes	0	N/A	Pos	Pos	
637	2	F	13	OS	D	79	3	3					No			Pos	Pos	
638	3	F	35	OD	D	77	0	0					Yes	0	pT1	Pos	Pos	
639	2	F	73	OD	D	6	5	0					No			Pos	Pos	
640	3	M	49	OD	D	74	0	0					Yes	0	pT1	Pos	Pos	
641	3	F	8	OS	D	73	0	0					Yes	0	pT3b	Pos	Pos	
642	1	M	16	OS	D	68	2	0	2	0	0	Yes	No			Pos	Pos	
643	1	M	27	OD	D	70	4	0	3	0	0	No	No			Pos	Pos	
644	2	F	28	OD	D	5	3	2					No			Pos	Pos	
645	2	M	23	OS	D	71	2	0					Yes	3	pT1	Pos	Pos	
646	2	F	30	OS	D	4	0	4					Yes	7	N/A	Pos	Pos	
647	1	M	30	OS	D	71	2	0	2	0	2	Yes	Yes	7	pT3d	Pos	Pos	
648	3	M	24	OS	D	71	0	0					Yes	0	pT1	Pos	Pos	

Safety of Tylectomy in Salvage of Eyes Affected by Retinoblastoma
Raw data, Table S2

Patient ID	Group	Sex	Age at Dx months	Studied Eye	IIRC	Follow-up Months	Pre-Tyl IVC #	Pre-Tyl IAC #	Dx to Tyl months	Post-Tyl IVC #	Post-Tyl Ivitc #	Post-Tyl Recur-rence	Enu	Dx to Enu	pTNM	OS	DDS	Dx to Death months
649	3	M	51	OS	D	64	0	0					Yes	0	N/A	Pos	Pos	
650	1	F	2	OS	D	70	2	0	2	0	2	No	No			Pos	Pos	
651	2	F	47	OD	D	67	0	4					No			Pos	Pos	
652	3	M	21	OS	D	66	0	0					Yes	0	pT3b	Pos	Pos	
653	3	M	14	OD	D	54	0	0					Yes	0	N/A	Pos	Pos	
654	3	F	10	OD	D	65	0	0					Yes	0	pt2a	Pos	Pos	
655	1	M	39	OD	D	64	0	3	4	0	0	No	No			Pos	Pos	
656	1	F	5	OS	D	63	2	0	2	0	1	No	No			Pos	Pos	
657	2	M	13	OD	D	64	0	4					Yes	5	N/A	Pos	Pos	
658	1	M	29	OD	D	64	4	0	5	0	2	Yes	Yes	13	pT3c	Pos	Pos	
659	3	M	3	OD	D	72	0	0					Yes	0	pT1	Pos	Pos	
660	1	F	9	OS	D	63	2	0	1	0	2	No	No			Pos	Pos	
661	2	M	20	OS	D	77	0	4					Yes	6	pT1	Pos	Pos	
662	1	M	30	OS	D	66	5	4	19	0	0	No	No			Pos	Pos	
663	2	M	19	OD	D	4	2	1					No			Pos	Pos	
664	2	F	21	OS	D	71	0	3					No			Pos	Pos	
665	3	M	11	OS	D	63	0	0					Yes	0	pT1	Pos	Pos	
666	2	M	36	OD	D	67	6	0					Yes	7	pT1	Pos	Pos	
667	1	M	36	OS	D	75	1	1	2	1	0	No	No			Pos	Pos	
668	2	M	23	OD	D	2	2	0					No			Pos	Pos	
669	2	F	2	OD	D	2	2	0					No			Pos	Pos	
670	1	M	21	OD	D	65	0	0	1	0	0	No	No			Pos	Pos	
671	1	F	5	OD	D	65	3	0	3	0	0	Yes	No			Pos	Pos	
672	3	M	10	OD	D	14	0	0					Yes	0	pT3b	Neg	Neg	14
673	2	F	21	OD	D	66	2	0					Yes	3	pT1	Pos	Pos	
674	3	M	22	OS	D	38	0	0					Yes	0	N/A	Pos	Pos	
675	3	F	57	OD	D	61	0	0					Yes	0	pT1	Pos	Pos	
676	2	M	33	OS	D	70	2	2					No			Pos	Pos	
677	1	M	43	OS	D	68	3	0	3	0	1	No	No			Pos	Pos	
678	2	M	25	OS	D	68	2	1					Yes	3	pT1	Pos	Pos	
679	2	F	21	OS	D	13	0	4					No			Pos	Pos	
680	1	F	42	OD	D	62	3	0	3	1	1	Yes	Yes	12	pT4	Pos	Pos	
681	1	M	5	OD	D	63	2	0	2	0	0	Yes	Yes	11	pT1	Pos	Pos	
682	1	M	31	OD	D	64	4	0	6	0	0	No	No			Pos	Pos	
683	2	M	3	OS	D	65	3	0					Yes	3	pT1	Pos	Pos	
684	2	F	28	OD	D	27	2	0					Yes	3	pT3b	Neg	Neg	27
685	1	F	45	OS	D	64	5	0	6	0	0	No	No			Pos	Pos	
686	2	M	62	OS	D	63	2	0					Yes	2	pT1	Pos	Pos	
687	1	F	4	OD	D	61	0	3	6	0	0	No	No			Pos	Pos	
688	2	M	9	OD	D	63	3	0					Yes	4	N/A	Pos	Pos	
689	3	M	11	OD	D	74	0	0					Yes	0	N/A	Pos	Pos	
690	2	F	25	OD	D	3	0	3					No			Pos	Pos	
691	2	M	20	OD	D	46	0	4					No			Pos	Pos	
692	3	F	19	OD	D	74	0	0					Yes	0	pT3b	Pos	Pos	
693	1	M	24	OS	D	66	0	2	3	0	1	No	No			Pos	Pos	
694	2	F	10	OD	D	74	2	0					Yes	3	pT1	Pos	Pos	
695	2	F	32	OS	D	58	2	0					Yes	2	pT3b	Pos	Pos	
696	2	F	23	OD	D	1	2	0					No			Pos	Pos	
697	1	F	36	OD	D	17	3	0	2	0	0	Yes	Yes	8	pT3a	Neg	Neg	17
698	2	M	12	OD	D	68	6	0					No			Pos	Pos	
699	2	M	19	OD	D	66	2	0					Yes	2	Pt3b	Pos	Pos	
700	1	M	26	OD	D	68	2	0	2	0	0	Yes	Yes	13	pT3d	Pos	Pos	
701	3	M	36	OS	D	62	0	0					Yes	0	pT3d	Pos	Pos	
702	1	M	15	OD	D	59	2	0	1	1	1	No	No			Pos	Pos	
703	1	F	3	OD	D	57	1	0	1	1	1	No	No			Pos	Pos	
704	3	M	23	OD	D	73	0	0					Yes	0	pT1	Pos	Pos	
705	1	M	27	OS	D	64	2	0	2	1	0	Yes	Yes	14	pT1	Pos	Pos	
706	2	F	30	OD	D	4	4	0					No			Pos	Pos	
707	1	M	33	OD	D	79	0	0	0	3	0	No	No			Neg	Pos	80
708	3	M	46	OD	D	59	0	0					Yes	0	pT1	Pos	Pos	
709	2	F	7	OS	D	1	2	0					No			Pos	Pos	
710	1	M	40	OD	D	56	1	0	1	1	1	No	No			Pos	Pos	
711	3	M	21	OS	D	57	0	0					Yes	0	pT1	Pos	Pos	
712	1	M	23	OD	D	61	1	0	1	1	0	No	No			Pos	Pos	
713	3	M	17	OD	D	56	0	0					Yes	1	pT1	Pos	Pos	
714	2	F	14	OD	D	55	1	0					Yes	1	pT1	Pos	Pos	
715	1	F	13	OS	D	61	2	0	2	1	0	No	No			Pos	Pos	
716	2	F	13	OS	D	61	3	0					Yes	2	pT1	Pos	Pos	
717	2	M	6	OS	D	58	3	3					No			Pos	Pos	
718	3	F	21	OS	D	65	0	0					Yes	0	pT1	Pos	Pos	
719	1	M	30	OD	D	61	3	0	3	1	0	No	No			Pos	Pos	
720	3	F	9	OD	D	34	0	0					Yes	0	N/A	Pos	Pos	

Safety of Tylectomy in Salvage of Eyes Affected by Retinoblastoma
Raw data, Table S2

Patient ID	Group	Sex	Age at Dx months	Studied Eye	IIRC	Follow-up Months	Pre-Tyl IVC #	Pre-Tyl IAC #	Dx to Tyl months	Post-Tyl IVC #	Post-Tyl Ivitc #	Post-Tyl Recur-rence	Enu	Dx to Enu	pTNM	OS	DDS	Dx to Death months
721	1	M	52	OD	D	61	3	0	4	0	0	Yes	Yes	15	pT1	Pos	Pos	
722	2	M	20	OS	D	63	3	0					Yes	2	pT1	Pos	Pos	
723	1	F	11	OD	D	60	0	1	1	1	0	No	No			Pos	Pos	
724	2	F	9	OS	D	38	4	0					Yes	9	pT1	Neg	Neg	38
725	3	M	12	OS	D	71	0	0					Yes	0	pT1	Pos	Pos	
726	1	F	36	OD	D	69	2	0	2	0	0	Yes	Yes	13	pT1	Pos	Pos	
727	2	M	3	OD	D	60	2	2					Yes	8	pT1	Pos	Pos	
728	2	F	36	OS	D	57	1	2					No			Pos	Pos	
729	3	F	41	OS	D	60	0	0					Yes	0	pT1	Pos	Pos	
730	1	M	4	OD	D	59	2	2	5	0	0	No	No			Pos	Pos	
731	3	F	41	OS	D	70	0	0					Yes	0	pT3a	Pos	Pos	
732	2	F	14	OD	D	63	4	0					Yes	9	pT1	Pos	Pos	
733	1	M	21	OD	D	60	6	0	6	1	0	No	No			Pos	Pos	
734	2	M	14	OD	D	57	2	3					No			Pos	Pos	
735	2	M	21	OS	D	36	6	3					No			Pos	Pos	
736	1	M	32	OD	D	62	2	2	5	0	0	No	No			Pos	Pos	
737	2	M	33	OD	D	58	2	0					Yes	3	pT1	Pos	Pos	
738	3	F	9	OD	D	0	0	0					Yes	0	pT2a	Pos	Pos	
739	3	M	22	OS	D	69	0	0					Yes	0	pT1	Pos	Pos	
740	1	F	45	OS	D	57	1	1	2	0	0	Yes	Yes	13	pT4	Pos	Pos	
741	3	F	34	OS	D	70	0	0					Yes	0	pT1	Pos	Pos	
742	1	M	5	OS	D	59	2	0	2	0	0	No	No			Pos	Pos	
743	1	M	65	OD	D	3	2	0	3	0	0	No	No			Pos	Pos	
744	3	M	25	OS	D	40	0	0					Yes	0	pT1	Pos	Pos	
745	1	M	10	OS	D	59	3	0	5	1	0	No	No			Pos	Pos	
746	3	M	44	OD	D	61	0	0					Yes	0	pT1	Pos	Pos	
747	2	F	31	OS	D	56	5	0					Yes	7	N/A	Pos	Pos	
748	2	F	4	OD	D	56	5	0					No			Pos	Pos	
749	2	F	1	OS	D	3	3	0					No			Pos	Pos	
750	2	M	80	OD	D	14	3	0					No			Pos	Pos	
751	1	F	37	OD	D	57	1	4	6	0	0	No	No			Pos	Pos	
752	1	F	24	OD	D	55	0	3	5	0	0	No	No			Pos	Pos	
753	3	M	29	OS	D	69	0	0					Yes	0	pT1	Pos	Pos	
754	2	M	23	OS	D	21	2	0					Yes	11	pT4	Neg	Neg	21
755	1	F	117	OD	D	67	0	6	45	0	1	Yes	Yes	59	pT2b	Pos	Pos	
756	1	M	30	OS	D	61	3	0	3	0	0	No	No			Pos	Pos	
757	1	F	25	OS	D	58	3	0	3	0	0	No	No			Pos	Pos	
758	1	M	17	OS	D	58	0	2	3	0	0	No	No			Pos	Pos	
759	2	M	43	OD	D	68	2	0					Yes	27	pT1	Pos	Pos	
760	2	M	4	OD	D	7	0	2					No			Pos	Pos	
761	1	M	13	OS	D	58	4	3	15	0	0	No	No			Pos	Pos	
762	3	M	32	OS	D	57	0	0					Yes	0	pT1	Pos	Pos	
763	2	M	21	OD	D	68	2	0					Yes	2	pT1	Pos	Pos	
764	2	F	5	OS	D	54	3	0					Yes	3	pT1	Pos	Pos	
765	1	F	37	OS	D	12	2	0	3	0	0	Yes	Yes	7	pT4	Neg	Neg	12
766	1	F	40	OD	D	60	2	0	2	0	0	Yes	Yes	20	pT2b	Pos	Pos	
767	2	M	19	OS	D	53	3	0					Yes	31	N/A	Pos	Pos	
768	2	M	41	OD	D	34		4					Yes	5	N/A	Pos	Pos	
769	1	M	10	OD	D	60	0	4	5	0	0	Yes	Yes	8	pT2b	Pos	Pos	
770	1	F	8	OD	D	52	2	0	2	0	0	No	No			Pos	Pos	
771	2	F	46	OS	D	54	2	3					No			Pos	Pos	
772	3	F	16	OD	D	54	0	0					Yes	0	pT1	Pos	Pos	
773	2	M	19	OS	D	2	0	2					No			Pos	Pos	
774	2	M	39	OS	D	2	0	2					No			Pos	Pos	
775	3	M	22	OD	D	56	0	0					Yes	0	pT1	Pos	Pos	
776	1	M	12	OD	D	58	3	0	3	1	0	No	No			Pos	Pos	
777	1	M	3	OS	D	59	3	0	3	1	0	No	No			Pos	Pos	
778	2	M	26	OS	D	53	2	4					No			Pos	Pos	
779	2	M	29	OS	D	3	4	0					No			Pos	Pos	
780	3	M	15	OS	D	56	0	0					Yes	0	pT1	Pos	Pos	
781	3	M	50	OS	D	66	0	0					Yes	0	pT1	Pos	Pos	
782	3	M	6	OD	D	58	0	0					Yes	0	pT1	Pos	Pos	
783	2	F	19	OD	C	87	2	0					No			Pos	Pos	
784	1	M	17	OS	E	82	4	0	7	0	1	Yes	Yes	14	pT1	Pos	Pos	
785	1	M	15	OD	C	79	3	2	6	0	0	Yes	No			Pos	Pos	
786	2	F	3	OD	C	27	10	0					No			Neg	Pos	27
787	2	F	13	OD	C	14	6	0					No			Pos	Pos	
788	1	M	19	OD	C	75	4	0	4	0	0	No	No			Pos	Pos	
789	2	M	12	OD	C	9	1	0					No			Neg	Neg	9
790	1	F	14	OD	C	71	2	0	17	0	0	No	No			Pos	Pos	
791	2	F	8	OD	C	59	5	0					No			Pos	Pos	
792	2	M	19	OD	C	18	2	0					No			Pos	Pos	

Safety of Tylectomy in Salvage of Eyes Affected by Retinoblastoma
Raw data, Table S2

Patient ID	Group	Sex	Age at Dx months	Studied Eye	IIRC	Follow-up Months	Pre-Tyl IVC #	Pre-Tyl IAC #	Dx to Tyl months	Post-Tyl IVC #	Post-Tyl Ivitc #	Post-Tyl Recur-rence	Enu	Dx to Enu	pTNM	OS	DDS	Dx to Death months
793	2	F	51	OD	C	55	6	0					No			Pos	Pos	
794	2	F	10	OD	C	54	2	0					No			Pos	Pos	
795	2	M	5	OD	C	59	4	2					No			Pos	Pos	
796	1	M	43	OS	D	78	4	0	4	2	0	Yes	No			Pos	Pos	
797	1	F	45	OS	D	79	5	0	5	0	1	No	No			Pos	Pos	
798	1	F	7	OD	C	82	6	1	15	0	0	No	No			Pos	Pos	
799	1	F	12	OS	D	71	3	0	3	1	0	No	No			Pos	Pos	
800	2	F	4	OD	C	5	5	0					No			Pos	Pos	
801	1	M	38	OD	C	75	3	0	4	0	0	No	No			Pos	Pos	
802	1	M	2	OS	D	68	3	0	4	0	1	No	No			Pos	Pos	
803	2	F	10	OD	C	14	4	3					No			Neg	Neg	15
804	2	M	11	OD	C	82	4	0					No			Pos	Pos	
805	2	M	11	OD	C	82	4	0					No			Pos	Pos	
806	1	F	8	OD	C	72	5	0	8	0	0	No	No			Pos	Pos	
807	2	F	32	OD	C	77	0	2					No			Pos	Pos	
808	1	M	6	OD	C	73	2	2	20	0	0	No	No			Pos	Pos	
809	1	M	4	OD	C	87	3	0	15	0	0	No	No			Pos	Pos	
810	1	F	1	OS	C	81	5	0	10	0	0	No	No			Pos	Pos	
811	2	M	14	OS	C	75	3	0					No			Pos	Pos	
812	1	F	49	OD	C	72	5	0	6	0	0	No	No			Pos	Pos	
813	1	M	21	OD	C	68	2	0	2	0	0	No	No			Pos	Pos	
814	1	F	24	OD	C	60	1	0	2	0	0	No	No			Pos	Pos	
815	1	M	9	OS	E	94	6	0	17	0	0	No	No			Pos	Pos	
816	2	F	12	OD	B	87	3	0					No			Pos	Pos	
817	1	M	1	OD	B	52	5	0	14	0	0	No	No			Pos	Pos	
818	1	F	4	OD	B	79	0	0	6	0	0	No	No			Pos	Pos	
819	2	M	10	OD	B	84	1	0					No			Pos	Pos	
820	2	F	2	OD	B	22	2	0					No			Pos	Pos	
821	2	F	4	OD	B	4	0	0					No			Pos	Pos	
822	1	F	9	OS	E	73	3	0	3	3	1	No	No			Pos	Pos	
823	2	F	2	OD	B	71	4	1					No			Pos	Pos	
824	2	F	2	OD	B	23	2	0					No			Neg	Pos	23
825	2	M	3	OD	B	4	4	0					No			Pos	Pos	
826	2	F	5	OD	B	74	5	0					No			Pos	Pos	
827	2	M	8	OD	B	68	5	0					No			Pos	Pos	
828	2	F	4	OD	B	72	1	0					No			Pos	Pos	
829	3	F	13	OS	E	12	0	0					Yes	0	N/A	Neg	Neg	12
830	2	M	3	OD	B	68	6	0					No			Pos	Pos	
831	2	F	5	OD	B	61	1	0					No			Pos	Pos	
832	2	M	3	OD	B	59	0	1					No			Pos	Pos	
833	2	F	4	OD	B	62	3	0					No			Pos	Pos	
834	1	F	7	OS	E	58	4	0	3	0	0	No	No			Pos	Pos	
835	2	M	14	OD	B	87	4	0					No			Pos	Pos	
836	2	F	6	OD	B	82	3	0					No			Pos	Pos	
837	1	F	33	OS	D	78	3	0	2	0	0	No	No			Pos	Pos	
838	1	M	27	OS	D	76	5	0	6	0	0	No	No			Pos	Pos	
839	2	F	6	OD	B	87	3	0					No			Pos	Pos	
840	2	M	5	OD	B	0	1	0					No			Pos	Pos	
841	1	F	6	OD	B	71	2	0	10	0	0	No	No			Pos	Pos	
842	2	F	7	OD	B	7	6	0					No			Pos	Pos	
843	1	F	11	OD	B	68	2	0	11	0	0	No	No			Pos	Pos	
844	2	M	3	OD	B	64	4	4					No			Pos	Pos	
845	1	F	13	OS	D	64	1	0	1	1	1	No	No			Pos	Pos	
846	2	F	2	OD	B	2	1	0					No			Pos	Pos	
847	2	F	4	OD	B	68	7	0					No			Pos	Pos	
848	2	M	25	OD	B	55	6	0					No			Pos	Pos	
849	1	F	14	OS	D	50	3	0	3	0	0	No	No			Pos	Pos	
850	2	M	5	OD	B	91	6	0					No			Pos	Pos	
851	2	M	2	OD	B	75	6	0					No			Pos	Pos	
852	2	M	2	OD	B	76	2	2					No			Pos	Pos	
853	2	M	0	OD	B	59	0	0					No			Pos	Pos	
854	2	M	26	OD	B	101	2	0					No			Pos	Pos	
855	2	M	0	OD	B	20	0	3					No			Neg	Pos	20
856	2	F	12	OD	B	11	0	0					No	0	N/A	Pos	Pos	
857	2	M	23	OD	B	61	4	0					No			Pos	Pos	
858	2	F	12	OS	B	81	0	0					No			Pos	Pos	
859	2	F	22	OS	B	85	0	0					No			Pos	Pos	
860	2	F	3	OS	B	71	1	0					No			Pos	Pos	
861	2	F	6	OS	B	72	0	0					No			Pos	Pos	
862	2	F	16	OS	B	73	2	0					No			Pos	Pos	
863	2	F	0	OS	B	64	2	0					No			Pos	Pos	
864	2	M	0	OS	B	70	0	0					No			Pos	Pos	

Safety of Tylectomy in Salvage of Eyes Affected by Retinoblastoma
Raw data, Table S2

Patient ID	Group	Sex	Age at Dx months	Studied Eye	IIRC	Follow-up Months	Pre-Tyl IVC #	Pre-Tyl IAC #	Dx to Tyl months	Post-Tyl IVC #	Post-Tyl Ivitc #	Post-Tyl Recur-rence	Enu	Dx to Enu	pTNM	OS	DDS	Dx to Death months
865	1	F	50	OS	B	64	1	0	3	0	0	No	No			Pos	Pos	
866	2	F	5	OS	B	9	3	0					No			Pos	Pos	
867	2	M	2	OD	B	2	0	0					No			Pos	Pos	
868	2	M	13	OD	A	89	0	0					No			Pos	Pos	
869	2	M	3	OD	A	78	0	0					No	14	N/A	Pos	Pos	
870	2	F	2	OS	E	27	3	0					No			Neg	Neg	27
871	2	M	4	OD	A	66	0	0					No	0	pT3b	Pos	Pos	
872	2	F	13	OD	A	64	2	0					No			Pos	Pos	
873	2	F	6	OD	A	83	0	0					No			Pos	Pos	
874	1	M	6	OS	D	64	2	0	2	0	0	No	No			Pos	Pos	
875	2	F	62	OD	A	87	0	0					No			Pos	Pos	
876	2	M	6	OD	A	58	6	0					No			Pos	Pos	
877	2	M	2	OD	A	73	0	0					No			Pos	Pos	
878	2	M	25	OD	A	77	0	0					No	0	N/A	Pos	Pos	
879	2	M	1	OD	A	87	0	0					No			Pos	Pos	
880	2	F	27	OS	D	15	8	0					No			Pos	Pos	
881	2	F	3	OS	D	48	7	1					No			Neg	Neg	48
882	1	F	4	OS	D	77	12	0	30	0	0	No	No			Pos	Pos	
883	2	F	9	OD	?	27	6	3					Yes	15	N/A	Pos	Pos	
884	2	M	8	OS	D	70	6	0					No			Pos	Pos	
885	1	M	35	OS	D	67	5	0	5	0	0	No	No			Pos	Pos	
886	2	M	4	OS	B	93	2	0					No			Pos	Pos	
887	2	M	2	OS	B	6	2	0					No			Pos	Pos	
888	2	M	25	OS	?	1	0	0					No	0	N/A	Pos	Pos	
889	1	M	19	OS	B	62	3	0	2	0	0	No	No			Pos	Pos	
890	3	M	44	OS	?	102	0	0					Yes	0	pT2b	Pos	Pos	
891	3	F	19	OD	?	100	0	0					Yes	0	N/A	Pos	Pos	
892	3	M	18	OD	?	88	0	0					Yes	0	N/A	Pos	Pos	
893	3	M	5	OD	?	11	0	0					Yes	0	N/A	Pos	Pos	
894	3	M	19	OS	?	93	0	0					Yes	0	pT2b	Pos	Pos	
895	2	M	10	OS	?	16	4	0					Yes	7	N/A	Pos	Pos	
896	3	F	4	OD	?	79	0	0					Yes	0	N/A	Pos	Pos	
897	3	M	25	OS	?	81	0	0					Yes	0	N/A	Pos	Pos	
898	3	M	25	OS	?	5	0	0					Yes	0	pT1	Pos	Pos	
899	2	M	30	OD	?	84	4	0					Yes	4	N/A	Pos	Pos	
900	3	F	2	OS	?	79	0	0					Yes	0	N/A	Pos	Pos	
901	3	M	25	OD	?	33	0	0					Yes	0	N/A	Pos	Pos	
902	3	F	7	OD	?	81	0	0					Yes	0	pT1	Pos	Pos	
903	3	M	28	OS	?	74	0	0					Yes	0	N/A	Pos	Pos	
904	3	F	28	OD	?	76	0	0					Yes	0	N/A	Pos	Pos	
905	3	M	55	OD	?	75	0	0					Yes	0	N/A	Pos	Pos	
906	3	F	28	OS	?	86	0	0					Yes	0	pT3a	Pos	Pos	
907	3	M	60	OD	?	72	0	0					Yes	0	N/A	Pos	Pos	
908	3	M	44	OS	?	48	0	0					Yes	0	N/A	Pos	Pos	
909	3	M	28	OS	?	69	0	0					Yes	0	N/A	Pos	Pos	
910	3	F	17	OS	?	71	0	0					Yes	0	N/A	Pos	Pos	
911	3	F	23	OS	?	80	0	0					Yes	0	Pt2a	Pos	Pos	
912	3	F	12	OS	?	47	0	0					Yes	0	N/A	Pos	Pos	
913	3	F	31	OD	?	78	0	0					Yes	0	N/A	Pos	Pos	
914	3	F	7	OS	?	3	0	0					Yes	0	pT1	Pos	Pos	
915	3	M	74	OS	?	0	0	0					Yes	0	N/A	Pos	Pos	
916	3	M	46	OD	?	76	0	0					Yes	0	N/A	Pos	Pos	
917	3	F	26	OS	?	64	0	0					Yes	0	N/A	Pos	Pos	
918	3	M	27	OS	?	66	0	0					Yes	0	N/A	Pos	Pos	
919	3	F	36	OS	?	69	0	0					Yes	0	N/A	Pos	Pos	
