# Supplementary material for: Tylectomy Safety in Salvage of Eyes with Retinoblastoma
Source: Cancers (Basel). 2021 Nov 22;13(22):5862. doi: 10.3390/cancers13225862 (PMC8616183; doi:10.3390/cancers13225862)
Supplement: Supplementary file 1 [file cancers-13-05862-s001.zip › Table S3.pdf]

Table S3. Treatments and outcomes of tumor recurrence in Group I

	Number of eyes/patients	Additional Tylectomy	Salvaged	Enucleated	Survived	Death	Disease-specific Death
**Recurrence after tylectomy**	75						
**Enucleation**	22				21	1	1
**Abandoned treatment**	1				0	1	1
**Additional salvage treatment**	52				43	9	7
**Laser or cryo**	23	0	11	12	20	3*	3
**Laser or cryo + IAC**	1	0	0	1	1	0	0
**Laser or cryo + IVitC**	2	0	1	1	2	0	0
**IAC**	1	0	0	1	1	0	0
**IVC**	4	0	1	3	3	1	1
**Tylectomy2**	14	14	8	6	12	2	2
**IVitC + Tylectomy2**	1	1	0	1	1	0	0
**IVC + Tylectomy2**	2	2	1	1	1	1	0
**Tylectomy3**	4	4	3	31	2	2	1
**TOTAL**	76	21	25	57	43	9	7

Cryo, cryotherapy; IAC, intra-arterial chemotherapy; IVitC, intravitreal chemotherapy; IVC, systemic chemotherapy

*Two patients refused enucleation despite tumor growth
